# Effects of Losartan on Patients Hospitalized for Acute COVID-19: A Randomized Controlled Trial

**DOI:** 10.1093/cid/ciae306

**Published:** 2024-07-10

**Authors:** Karen C Tran, Pierre Asfar, Matthew Cheng, Julien Demiselle, Joel Singer, Terry Lee, David Sweet, John Boyd, Keith Walley, Greg Haljan, Omar Sharif, Guillaume Geri, Johann Auchabie, Jean-Pierre Quenot, Todd C Lee, Jennifer Tsang, Ferhat Meziani, Francois Lamontagne, Vincent Dubee, Sigismond Lasocki, Daniel Ovakim, Gordon Wood, Alexis Turgeon, Yves Cohen, Eddy Lebas, Marine Goudelin, David Forrest, Alastair Teale, Jean-Paul Mira, Robert Fowler, Nick Daneman, Neill K J Adhikari, Marie Gousseff, Pierre Leroy, Gaetan Plantefeve, Patrick Rispal, Roxane Courtois, Brent Winston, Steve Reynolds, Peter Birks, Boris Bienvenu, Jean-Marc Tadie, Jean-Philippe Talarmin, Severine Ansart, James A Russell, for the ARBs CORONA II Team, J Russell, K Tran, M Cheng, P Asfar, J Demiselle, J Singer, P Mann, F Jain, K Tran, K Donohoe, V Leung, T Lee, K Tran, J Boyd, K Walley, K Tran, D Sweet, G Haljan, O Sharif, D Ovakim, G Wood, D Forrest, A Teale, S Reynolds, P Birk, B Winston, R Fowler, N Dameman, N Adhikari, J Tsang, M Cheng, F Lamontagne, A Turgeon-Fournier, Asfar, Demiselle, D G Geri, J Auchabie, J P Quenot, F Meziani, V Dubee, S Lasocki, Y Cohen, E Lebas, M Goudelin, J P Mira, M Gousseff, P Leroy, G Plantefev, P Rispal, R Courtois, B Bievenue, J M Tadie, J P Talarmin, S Ansart, Tae Won Yi, Adeera Levin

**Affiliations:** Division of General Internal Medicine, Vancouver General Hospital, University of British Columbia, Vancouver, Canada; Service de Médecine Intensive-Réanimation, Centre Hospitalier Universitaire d’Angers, Angers, France; McGill’s Interdisciplinary Initiative in Infection and Immunity, Divisions of Infectious Diseases and Medical Microbiology, McGill University Health Centre, Montreal, Quebec, Canada; Service de Médecine Intensive-Réanimation, Nouvel Hôpital Civil, Hôpitaux Universitaires de Strasbourg, Strasbourg, France; Centre for Health Evaluation and Outcome Science, St Paul's Hospital and University of British Columbia, Vancouver, Canada; Centre for Health Evaluation and Outcome Science, St Paul's Hospital and University of British Columbia, Vancouver, Canada; Division of General Internal Medicine, Vancouver General Hospital, University of British Columbia, Vancouver, Canada; Division of Critical Care Medicine, and Centre for Heart Lung Innovation, St Paul's Hospital, Vancouver, Canada; Division of Critical Care Medicine, and Centre for Heart Lung Innovation, St Paul's Hospital, Vancouver, Canada; Department of Medicine and Critical Care Medicine, Surrey Memorial Hospital, British Columbia, Canada; Department of Medicine and Critical Care Medicine, Surrey Memorial Hospital, British Columbia, Canada; Service de Médecine Intensive-Réanimation, Assistance Publique–Hôpitaux de Paris Ambroise Paré, Boulogne, France; Service de Réanimation Polyvalente, Centre Hospitalier de Cholet; Service de Médecine Intensive-Réanimation, Centre Hospitalier Universitaire Dijon, Dijon, France; McGill's Interdisciplinary Initiative in Infection and Immunity, McGill University Health Centre, Montreal, Quebec, Canada; Niagara Health, McMaster University, St Catherines, Ontario, Canada; Service de Médecine Intensive-Réanimation, Nouvel Hôpital Civil Strasbourg, Strasbourg, France; Centre Hospitalier Universitaire de Sherbrooke, University of Sherbrooke, Quebec, Canada; Service de Maladies Infectieuses, Centre Hospitalier Universitaire d'Angers, Angers, France; Service de Réanimation Chirurgicale, Centre Hospitalier Universitaire Angers, Angers, France; Royal Jubilee Hospital, Island Health, Victoria, British Columbia; Royal Jubilee Hospital, Island Health, Victoria, British Columbia; Department of Medicine, Centre Hospitalier Universitaire de Québec–Université Laval, Quebec, Canada; Service de Médecine Intensive-Réanimation, Assistance Publique–Hôpitaux de Paris Avicenne, Bobigny, France; Service de Réanimation Polyvalente, Centre Hospitalier Bretagne-Atlantique, Vannes, France; Service de Réanimation Polyvalente, Centre Hospitalier Universitaire Limoges, Limoges, France; Department of Medicine, Nanaimo Regional General Hospital, British Columbia, Canada; Department of Medicine, Nanaimo Regional General Hospital, British Columbia, Canada; Service de Médecine Intensive-Réanimation, Assistance Publique-Hôpitaux de Paris, Cochin, France; Critical Care Medicine, Sunnybrook Health Sciences Centre, Toronto, Ontario, Canada; Critical Care Medicine, Sunnybrook Health Sciences Centre, Toronto, Ontario, Canada; Critical Care Medicine, Sunnybrook Health Sciences Centre, Toronto, Ontario, Canada; Service de Médecine Interne–Maladies Infectieuses–Hématologie, Centre Hospitalier Bretagne-Atlantique, Vannes, France; Service de médecine polyvalente et maladies infectieuses, Centre Hospitalier Melun, Melun, France; Service de Réanimation Polyvalente, Centre Hospitalier Argenteuil, France; Department of Medicine, Service de médecine interne, Centre Hospitalier Agen, Agen, France; Service de Médecine post-urgences–Maladies infectieuses, Centre Hospitalier de Cholet, Cholet, France; Departments of Critical Care Medicine, Medicine, and Biochemistry and Molecular Biology, Foothills Medical Centre, University of Calgary, Alberta, Canada; Critical Care Medicine, Royal Columbian Hospital, New Westminster, British Columbia, Canada; Department of Medicine, Simon Fraser University, Surrey, British Columbia, Canada; Critical Care Medicine, Royal Columbian Hospital, New Westminster, British Columbia, Canada; Department of Medicine, Simon Fraser University, Surrey, British Columbia, Canada; Service de médecine interne, Hôpital St Joseph, Marseille, France; Service de Médecine Intensive-Réanimation et de Maladies Infectieuses, Centre Hospitalier Universitaire de Rennes, Rennes, France; Service de médecine interne, maladies du sang et infectiologie, Centre Hospitalier de Quimper, Quimper, France; Service de Maladies Infectieuses, Centre Hospitalier Régional Universitaire Brest, Brest, France; Division of Critical Care Medicine, and Centre for Heart Lung Innovation, St Paul's Hospital, Vancouver, Canada

**Keywords:** COVID-19, losartan, angiotensin receptor blocker, mortality

## Abstract

**Background:**

Severe acute respiratory syndrome coronavirus 2 (SARS-CoV-2) down-regulates angiotensin-converting enzyme 2, potentially increasing angiotensin II. We hypothesized that losartan compared to usual care decreases mortality and is safe in patients hospitalized with coronavirus disease 2019 (COVID-19). We aimed to evaluate the effect of losartan versus usual care on 28-day mortality in patients hospitalized for acute COVID-19.

**Methods:**

Eligibility criteria included adults admitted for acute COVID-19. Exclusion criteria were hypotension, hyperkalemia, acute kidney injury, and use of angiotensin receptor blockers (ARBs) or angiotensin-converting enzyme inhibitors within 7 days. Participants were randomized to losartan 25–100 mg/day orally for the hospital duration or 3 months or the control arm (usual care) in 29 hospitals in Canada and France. The primary outcome was 28-day mortality. Secondary outcomes were hospital mortality, organ support, and serious adverse events (SAEs).

**Results:**

The trial was stopped early because of a serious safety concern with losartan. In 341 patients, any SAE and hypotension were significantly higher in the losartan versus usual care groups (any SAE: 39.8% vs 27.2%, respectively, *P* = .01; hypotension: 30.4% vs 15.3%, respectively, *P* < .001) in both ward and intensive care patients. The 28-day mortality did not differ between losartan (6.5%) versus usual care (5.9%) (odds ratio, 1.11 [95% confidence interval, .47–2.64]; *P* = .81), nor did organ dysfunction or secondary outcomes.

**Conclusions:**

Caution is needed in deciding which patients to start or continue using ARBs in patients hospitalized with pneumonia to mitigate risk of hypotension, acute kidney injury, and other side effects. ARBs should not be added to care of patients hospitalized for acute COVID-19.

**Clinical Trials Registration:**

NCT04606563.

## Teaser

Angiotensin receptor blockers (ARBs) should not be used for the treatment of acute COVID-19 infections due to safety concerns. ARBs should be used cautiously in patients hospitalized with pneumonia.

Coronavirus disease 2019 (COVID-19) has led to many hospitalizations, intensive care unit (ICU) admissions, and need for life support. Randomized controlled trials (RCTs) of dexamethasone [1], prophylactic anticoagulation [2], and immune regulators (interleukin 6 receptor antagonists, eg, tocilizumab [3]) and Janus kinase inhibitors (eg, baricitinib [4]) for patients hospitalized for acute COVID-19 have shown decreased need for ventilation and mortality rates.

Severe acute respiratory syndrome coronavirus 2 (SARS-CoV-2), the viral cause of COVID-19 [5], down-regulates angiotensin-converting enzyme 2 (ACE2), increasing angiotensin II [6] and promoting lung injury [7], risk of acute respiratory distress syndrome, cardiac injury [8], inflammation, coagulation, endothelial dysfunction [9], tissue factor and plasminogen activator inhibitor-1 expression [10], and microvascular thrombosis. The angiotensin II receptor 1 blocker losartan decreases tissue factor [11], plasminogen activator inhibitor 1 [10, 11], vascular inflammation, and oxidative stress.

Thus, angiotensin receptor blockers (ARBs) are rational therapy for acute COVID-19 [12], blocking angiotensin II effects. Angiotensin II is hydrolyzed to angiotensin-(1-7) by the ACE2 pathway; angiotensin-(1-7) induces vasodilation and anti-inflammatory actions. The binding of SARS-CoV-2 to ACE2 may prevent ACE2 conversion of angiotensin II to angiotensin-(1-7), leading to unopposed angiotensin II. Losartan limits lung injury and decreases viral titer in murine influenza A(H7N9) [13].

Trials of ARBs in patients not previously on ARBs [14–17] and trials of continuing versus discontinuing ARBs [18–20] in patients with acute COVID-19 generally found no difference in mortality [21].

In this randomized open-label trial of losartan versus usual care, our primary hypothesis was that losartan compared to usual care decreases 28-day mortality and is safe in adults not previously on ARBs or angiotensin-converting enzyme (ACE) inhibitors who were hospitalized for acute COVID-19.

## METHODS

### Study Design

This is a prospective, open-label, randomized trial that randomized patients hospitalized between 20 October 2020 and 1 March 2022 with acute COVID-19 to losartan or usual care in 29 hospitals in Canada and France. This research protocol was approved by the research ethics boards of each institution. The study protocol is found in the Supplementary Appendix. The trial was registered at ClinicalTrials.gov (NCT04606563).

### Participants

Inclusion criteria were adults ≥18 years of age who were admitted for the first time for acute COVID-19 who had laboratory-proven SARS-CoV-2 within 14 days prior to hospital admission. Exclusion criteria were hypotension (systolic arterial pressure <100 mm Hg, diastolic arterial pressure <50 mm Hg or mean arterial pressure <65 mm Hg), hyperkalemia (potassium >5.5 mmol/L), acute kidney injury (urine output <0.5 mL/kg/hour and new creatinine >200 µmol/L, or increase >100 µmol/L, or glomerular filtration rate <30 mL/minute), use of ARBs or ACE inhibitors within 7 days of presentation, pregnant or breastfeeding, known allergy to ARBs, use of aliskiren in patients with diabetes mellitus or moderate to severe renal impairment (glomerular filtration rate <60 mL/minute), and a “do not resuscitate” order (patients not wishing to receive cardiopulmonary resuscitation, but could participate if other medical treatments would be given). Informed consent was obtained from all patients or their next of kin if they were unable to provide consent.

### Randomization and Masking

Each patient was randomized using permuted blocks of random size 2 and 4 to losartan or usual care and stratified by site (details in Supplementary Appendix). Clinicians, investigators, and research staff were blinded to randomization sequence, but treatment was open-label (Supplementary Appendix).

### Procedures

Losartan dosing is 25–100 mg daily for other conditions, so a 100 mg maximum losartan dose was chosen to optimize efficacy and safety. Patients were randomized, stratified by site, within 72 hours to oral losartan 25–100 mg/day, up-titrating based on treatment tolerance or usual care for up to 3 months if still hospitalized. Crossover and open-label losartan were not permitted. Subjects could have losartan discontinued if they developed a serious adverse event (SAE). Patients were followed at 1, 3, and 6 months postrandomization.

### Outcomes

The primary outcome was 28-day mortality. The secondary outcomes were hospital mortality; ICU admission (if not in ICU when randomized) and days alive and free (DAF) of ICU during first 28 days; hospital length of stay; use of and DAF of vasopressors, invasive ventilation, and renal replacement therapy (RRT); acute cardiac injury (definition: increased troponin and/or N-terminal pro-brain natriuretic peptide relative to baseline and use of inotropic agents); SAEs; 1-, 3-, and 6-month mortality; and cardiac, pulmonary, coagulation, and renal function. Research biomarker studies were planned (Supplementary Appendix Text 2).

Pooled safety endpoints and SAEs were reported to (*i*) 2 independent medical monitors who adjudicated the sites’ judgment regarding SAEs and relatedness to losartan and (*ii*) an independent data and safety monitoring committee (DSMC). Predefined SAEs were hypotension (systolic arterial pressure <90 mm Hg or mean arterial pressure <65 mm Hg requiring fluid resuscitation or use of vasopressors), hyperkalemia (potassium >5.5 mmol/L), acute kidney injury (urine output <0.5 mL/kg/hour or new serum creatinine >200 µmol/L or increase of serum creatinine of >100 µmol/L or 2-fold increase in baseline serum creatinine) and severe hypersensitivity reactions. SAEs were reported within 24 hours to the Coordinating Centre, regulatory authorities (Health Canada), and local research ethics boards.

Data according to treatment group were referred to the DSMC that planned for safety analyses after 25%, 50%, and 75% of patient recruitment and on an ad hoc basis if serous concerns arose. Three interim analyses and 1 final analysis were planned as per Peto (*P* < .001 at any analysis; *P* = .049 for final analysis for mortality increase in either group). The DSMC had a charter (Supplementary Appendix) and could use its discretion in recommending study termination to the Steering Committee.

### Statistical Analysis

At the time of trial design, the mortality rate of patients hospitalized with acute COVID-19 was 17% in publications of 18 709 hospitalized COVID-19 patients [22] and 15% in our ARBs CORONA I cohort [23]. To detect 5% absolute 28-day mortality rate difference, assuming 10% losartan versus 15% usual care mortality (80% power, 2-tailed α = .05) based on χ^2^ test and allowing a 10% contingency for participants wishing to discontinue, a sample size of 1372 was required.

For binary outcomes, χ^2^ test or Fisher exact test was used to compare losartan versus usual care. Logistic regression was used to express the difference between groups’ odds ratio (OR) with 95% confidence interval (CI). Time to event variable was compared using Kaplan-Meier curves with log-rank test. Time to hospital discharge was censored at day 90 for patients hospitalized for >90 days and all in-hospital deaths were considered never discharged and censored at day 91. Difference between groups for time to death was expressed as hazard ratio (HR) using Cox regression. For time to hospital discharge, the proportional hazard assumption cannot be satisfied and censored quartile regression was used. DAF of ICU was compared by Wilcoxon rank-sum test. Patients who died within 28 days were assigned a value of 0 to increase penalty of death. Supplementary regression analysis (logistic, Cox, censored quantile or quantile) adjusting for predefined factors (age, sex, and comorbidities [hypertension, diabetes mellitus, cardiovascular disease, and chronic kidney disease]) were performed. Firth penalized method was applied in the logistic and Cox regression analysis due to low event rates. Comparison of DAF of vasopressors, invasive ventilation, and RRT during the first 14 days between groups was by Wilcoxon rank-sum test. Patients who died within 14 days were assigned a value of 0. Patients discharged before day 14 were assumed to be free of organ support after discharge. Adjusted regression analysis for DAF of organ support as count data could not be performed without numerical issues as few patients had non-zero and non-14 DAF. Instead, adjusted analysis for the binary outcome DAF <14 days was examined. All *P* values and CIs were not adjusted for multiple testing.

### Subgroups

Losartan versus usual care was compared in subgroups defined by ICU admission status on randomization day and (*i*) sex, (*ii*) ethnicity, (*iii*) homeless and underhoused populations, (*iv*) hypertension, (*v*) heart failure, (*vi*) chronic kidney disease, and (*vii*) diabetes, by including an interaction term between subgroup variable and treatment group in the aforementioned adjusted regression models.

## RESULTS

A Randomized, Embedded, Multi-factorial, Adaptive Platform Trial for Community-Acquired Pneumonia (REMAPCAP), a randomized trial of ARBs or ACE inhibitors versus usual care in patients hospitalized for acute COVID-19, was stopped early after a planned interim analysis because of significantly increased mortality and acute kidney injury rates in the ARB arm compared to usual care among the critically ill patient stratum.

The REMAPCAP trial led us to pause ARBs CORONA II, do an unplanned interim analysis (albeit only 2 patients short of the first planned interim analysis), and report that to the ARBs CORONA II DSMC. The DSMC recommended a futility analysis that showed 7% chance of a statistically significant result in either direction, assuming enrollment of non–critically ill patients whose expected mortality was 1.875% and 1.25% in the 2 arms (Supplementary Text 4). The DSMC unanimously recommended stopping ARBs CORONA II.

**Figure 1.**
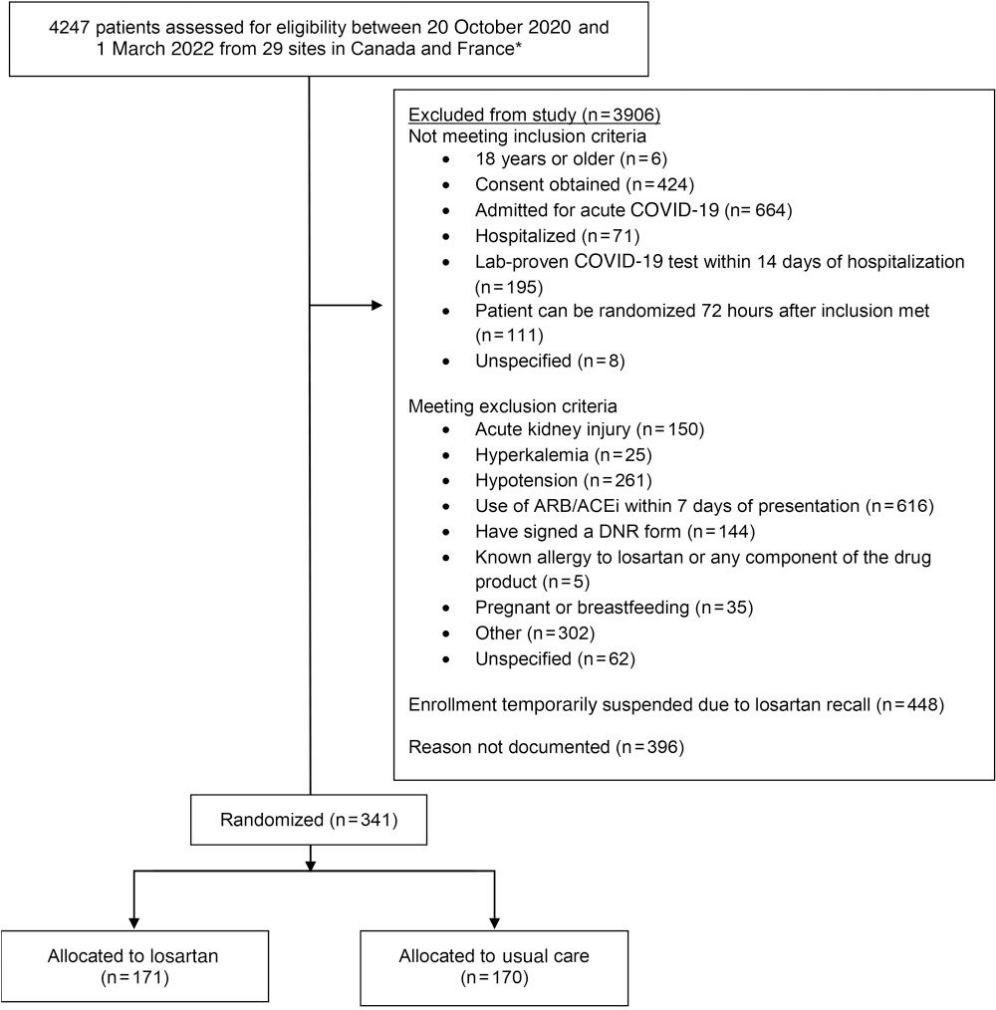
Study flow diagram of the ARBs CORONA II trial of losartan versus usual care in patients hospitalized for acute coronavirus disease 2019, including inclusions, exclusions, reasons for exclusions, and final numbers included in the treatment and control groups. *see supplement for list of sites. Abbreviations: ACEi, angiotensin-converting enzyme inhibitor; ARB, angiotensin receptor blocker; COVID-19, coronavirus disease 2019; DNR, do not resuscitate.

Patient flow is shown in Figure 1. Patients were enrolled from 20 October 2022 to 1 March 2022 (Supplementary Table 1).

Patients were well-matched for age, sex, and comorbidities (Table 1, Supplementary Tables 1 and 2). Preenrollment vaccination rates were low (losartan 17%; usual care 20.9%). More than half of patients were admitted to ICU at admission. In the losartan arm, 65% of patients were treated with 100 mg, the maximum dose.

**Table 1.** Baseline Characteristics of Patients Randomized to Losartan Versus Usual Care in the ARBs CORONA II Trial of Losartan Versus Usual Care in Patients Hospitalized for Acute Coronavirus Disease 2019

Variable	All (N = 341)	Losartan (n = 171)	Usual Care (n = 170)
Randomization date, No. (%)			
2020 Q4	6 (1.8)	3 (1.8)	3 (1.8)
2021 Q1	43 (12.6)	23 (13.5)	20 (11.8)
2021 Q2	160 (46.9)	78 (45.6)	82 (48.2)
2021 Q3	84 (24.6)	43 (25.1)	41 (24.1)
2021 Q4	26 (7.6)	12 (7.0)	14 (8.2)
2022 Q1	22 (6.5)	12 (7.0)	10 (5.9)
Country, No. (%)			
Canada	140 (41.1)	70 (40.9)	70 (41.2)
France	201 (58.9)	101 (59.1)	100 (58.8)
Age, y, mean (SD)	56.4 (13.5)	57.4 (13.2)	55.3 (13.8)
Sex, No. (%)			
Female	102 (29.9)	54 (31.6)	48 (28.2)
Male	239 (70.1)	117 (68.4)	122 (71.8)
Ethnic group, No. (%)			
Unknown	116	57	59
White	133 (59.1)	72 (63.2)	61 (55.0)
Black	9 (4.0)	4 (3.5)	5 (4.5)
Latino	5 (2.2)	2 (1.8)	3 (2.7)
Middle Eastern	36 (16.0)	15 (13.2)	21 (18.9)
South Asian	6 (2.7)	4 (3.5)	2 (1.8)
Southeast Asian	14 (6.2)	10 (8.8)	4 (3.6)
East Asian	15 (6.7)	6 (5.3)	9 (8.1)
Indigenous	3 (1.3)	0 (0.0)	3 (2.7)
Other	4 (1.8)	1 (0.9)	3 (2.7)
Homeless, No. (%)	4 (1.2)	3 (1.8)	1 (0.6)
COVID-19 vaccination prior to day 0, No. (%)			
Any	52/275 (18.9)	24/141 (17.0)	28/134 (20.9)
2 doses (or 1 dose of Janssen vaccine)	26/276 (9.4)	15/142 (10.6)	11/134 (8.2)
Comorbidities, No. (%)			
Hypertension	65 (19.2)	35 (20.7)	30 (17.6)
Chronic cardiac disease	26 (7.6)	16 (9.4)	10 (5.9)
Chronic kidney disease	9 (2.6)	3 (1.8)	6 (3.5)
Diabetes	62 (18.2)	26 (15.2)	36 (21.2)
Chronic pulmonary disease (not asthma)	17 (5.0)	9 (5.3)	8 (4.7)
Asthma (physician diagnosed)	26 (7.6)	13 (7.6)	13 (7.6)
Liver disease	3 (0.9)	1 (0.6)	2 (1.2)
Prior stroke	6 (1.8)	3 (1.8)	3 (1.8)
Dementia	5 (1.5)	2 (1.2)	3 (1.8)
Other chronic neurological disorder	15 (4.4)	8 (4.7)	7 (4.1)
Malignant neoplasm	13 (3.8)	8 (4.7)	5 (3.0)
Chronic hematologic disease	9 (2.6)	6 (3.5)	3 (1.8)
HIV/AIDS	2 (0.6)	1 (0.6)	1 (0.6)
Hypercholesterolemia	33 (9.7)	11 (6.4)	22 (13.0)
Rheumatologic disorder	9 (2.6)	4 (2.3)	5 (3.0)
Venous thromboembolism	10 (2.9)	6 (3.5)	4 (2.4)
Smoking, No. (%)	16 (4.9)	8 (4.9)	8 (4.9)
BMI, kg/m^2^, mean (SD)	30.4 (6.4)	29.9 (6.7)	30.9 (6.0)
Temperature, ^°^C, mean (SD)	37.2 (0.9)	37.1 (0.9)	37.2 (0.9)
Heart rate, beats/min, mean (SD)	84.2 (16.6)	83.0 (16.3)	85.4 (16.8)
Respiratory rate, breaths/min, mean (SD)	25.9 (6.5)	25.4 (6.4)	26.4 (6.5)
Oxygen saturation, %, mean (SD)	93.4 (3.7)	93.5 (3.8)	93.2 (3.7)
On oxygen therapy, No. (%)	310/340 (91.2)	153/170 (90.0)	157/170 (92.4)
PaO_2_/FiO_2_, median (IQR)	120.0 (83.1–163.3)	114.0 (76.0–152.3)	120.0 (84.3–177.5)
Bilirubin, μmol/L, median (IQR)	8.0 (6.4–11.0)	8.0 (6.0–11.0)	8.0 (6.6–11.0)
Creatinine, μmol/L, median (IQR)	67.0 (56.3–79.0)	69.0 (55.0–80.0)	66.5 (57.0–77.0)
Ferritin, ng/mL, median (IQR)	1154 (641–1693)	1200 (465–1909)	1063.5 (686–1562)
Hemoglobin, g/L, median (IQR)	135 (124–144)	135.5 (123–144)	135 (126–144)
Platelet count, ×10^9^/L, median (IQR)	228 (176–299)	223 (163–299)	232 (188–296)
WBC count, ×10³/μL, median (IQR)	6.9 (4.8–9.4)	7.1 (4.6–9.2)	6.9 (5.0–9.6)
CRP, μg/mL, median (IQR)	73.7 (43.0–129.0)	71.7 (43.0–122.0)	74.5 (42.8–132.0)
Mean arterial pressure, mm Hg, median (IQR)	83 (74–92)	84 (75–92)	82 (73–93)
NT-pro-BNP, median (IQR)	125 (49–350)	137.5 (50–426)	95 (40–282)
D-dimer level, ng/mL, median (IQR)	804 (527–1480)	861 (540–1419)	792 (514–1490)
INR, median (IQR)	1.10 (1.00–1.16)	1.10 (1.00–1.18)	1.10 (1.00–1.13)
Troponin, ng/mL, median (IQR)	0.0080 (0.0050–0.0159)	0.0080 (0.0050–0.0170)	0.0080 (0.0040–0.0140)
Glasgow Coma Scale score, No. (%)			
Unknown	124	62	62
13–15	196 (90.3)	98 (89.9)	98 (90.7)
9–12	2 (0.9)	1 (0.9)	1 (0.9)
≤8	19 (8.8)	10 (9.2)	9 (8.3)
In ICU on day 0, No. (%)	187 (54.8)	93 (54.4)	94 (55.3)
Organ support on day 0, No. (%)			
Vasopressors	14 (4.1)	6 (3.5)	8 (4.7)
Ventilation	102 (30.0)	51 (30.0)	51 (30.0)
Invasive ventilation	48 (14.1)	21 (12.4)	27 (15.9)
Noninvasive ventilation	61 (17.9)	32 (18.8)	29 (17.1)
RRT	1 (0.3)	1 (0.6)	0 (0.0)
ECMO	1 (0.3)	1 (0.6)	0 (0.0)
COVID-19 severity: modified 4C Mortality Score^a^, median (IQR)	6 (3–8)	6 (4–8)	6 (3–8)

Abbreviations: BMI, body mass index; COVID-19, coronavirus disease 2019; CRP, C-reactive protein; ECMO, extracorporeal membrane oxygenation; HIV, human immunodeficiency virus; ICU, intensive care unit; INR, international normalized ratio; IQR, interquartile range; NT-pro-BNP, N-terminal pro-brain natriuretic peptide; PaO_2_/FiO_2_, partial pressure of oxygen/fraction of inspired oxygen; RRT, renal replacement therapy; SD, standard deviation; WBC, white blood cell.

^a^COVID-19 severity was based on a modified version of the 4C Mortality Score [24]. The total of the modified 4C Mortality Score ranged from 0 to 17. A higher score predicts a higher chance of dying during hospitalization. In the primary publication, the score includes age, sex, comorbidities, respiratory rate, oxygen saturation, Glasgow Coma Scale (GCS), and urea and CRP. GCS and CRP were excluded from our calculation of a modified 4C Mortality Score because data were not consistently captured. Definition of comorbidities was based on the predefined items in ARBs CORONA II (see table) instead of those defined by the Charlson Comorbidity Index as used in the original 4C Mortality Score. Urea was not captured in our study and so serum creatinine level was used to measure renal function at 3 levels comparable to urea as follows: normal, <110 μmol/L; moderate, 110–220 μmol/L; more than moderate, >220 μmol/L. We previously validated a similar version of the modified score (with a slightly different set of predefined comorbidities) using data from ARBs CORONA I in which it had an area under the receiver operating characteristic curve of 0.76 for in-hospital mortality similar to that in the original paper, in which it was 0.79.

All treated patients received losartan (a later protocol amendment to allow use of any ARB was not implemented because of the early cessation of the trial). Of 171 losartan-treated patients, losartan was discontinued in 34 (20.2%) for SAEs, in 10 (6%) at patient request, in 5 (3%) for development of a medical condition of substantial risk, and in 16 (9.5%) for other reasons (Supplementary Table 1).

**Figure 2.**
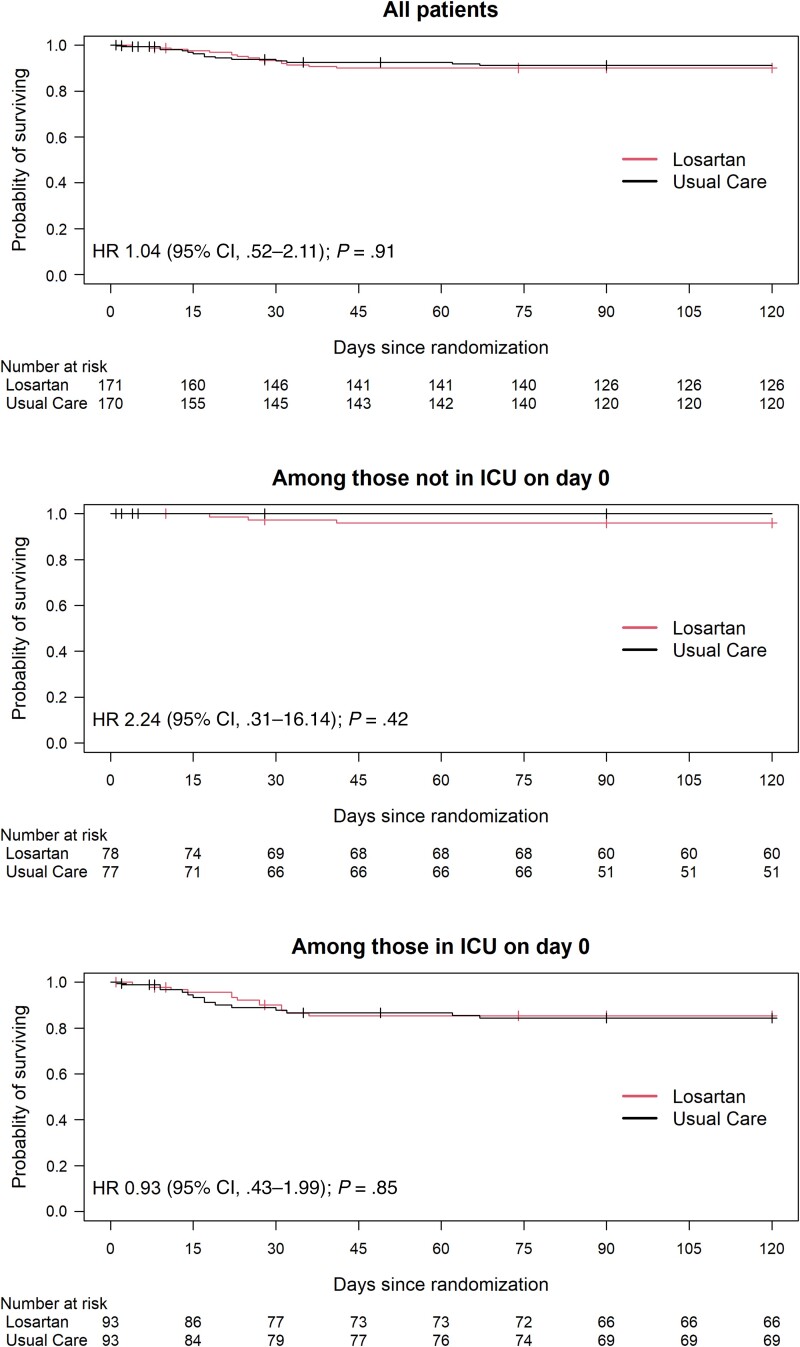
Kaplan-Meier curves for time to death of patients (all patients, those not in intensive care unit [ICU], and those in ICU on day of admission to study) of the ARBs CORONA II trial of losartan versus usual care in patients hospitalized for acute coronavirus disease 2019. There were no differences in time to death overall or in ICU-admitted versus not ICU-admitted subgroups. Abbreviations: CI, confidence interval; HR, hazard ratio; ICU, intensive care unit.

The primary outcome—28-day mortality—was not different between losartan (6.5%) versus usual care (5.9%) (OR, 1.11 [95% CI, .47–2.64]; *P* = .81; Table 2, Figure 2), nor did 90- or 180-day mortality differ (Table 2).

**Table 2.** Mortality Rates and Secondary Outcomes in the ARBs CORONA II Trial of Losartan Versus Usual Care in Acute Coronavirus Disease 2019 (COVID-19) of All Patients and According to Losartan Versus Usual Care Arm in Patients Hospitalized for Acute COVID-19

Variable	Losartan (n = 171)	Usual Care (n = 170)	Estimated Effect (95% CI)^a^	*P* Value
Mortality, No. (%)^b^				
28-day				
All patients	11/168 (6.5)	10/169 (5.9)	1.11 (.47–2.64)	.81
Among non–critically ill patients on day 0	2/76 (2.6)	0/77 (0.0)	5.20 (.24–112.36)	.29
Among critically ill patients on day 0	9/92 (9.8)	10/92 (10.9)	0.89 (.34–2.30)	.81
90-day	16/168 (9.5)	14/169 (8.3)	1.16 (.55–2.44)	.69
180-day	16/168 (9.5)	16/169 (9.5)	1.01 (.49–2.07)	.99
In-hospital mortality, No. (%)	15/167 (9.0)	13/169 (7.7)	1.18 (.55–2.53)	.67
Organ support on first 14 d, No. (%)				
Vasopressors	38/170 (22.4)	31/170 (18.2)	1.29 (.76–2.18)	.35
Oxygen therapy	149/170 (87.6)	144/170 (84.7)	1.28 (.69–2.38)	.43
Ventilation	76/170 (44.7)	75/170 (44.1)	1.02 (.67–1.57)	.91
Invasive ventilation	49/170 (28.8)	48/168 (28.6)	1.01 (.63–1.62)	.96
Noninvasive ventilation	54/170 (31.8)	45/168 (26.8)	1.27 (.80–2.04)	.32
RRT	5/170 (2.9)	2/170 (1.2)	2.24 (.49–10.19)	.45
ECMO	4/170 (2.4)	4/170 (2.4)	1.00 (.25–4.07)	1.00
DAF first 14 d, median (IQR)^c^				
Vasopressors	14 (14–14)	14 (14–14)	0.0 (NC)	.36
Invasive ventilation	14 (9–14)	14 (10–14)	0.0 (NC)	.92
RRT	14 (14–14)	14 (14–14)	0.0 (NC)	.80
DAF first 14 d <14, No. (%)^c^				
Vasopressors	39 (22.9)	32 (18.8)	1.28 (.76–2.16)	.35
Invasive ventilation	50 (29.4)	49 (28.8)	1.03 (.64–1.64)	.91
RRT	8 (4.7)	7 (4.1)	1.14 (.42–3.13)	.79
Ever admitted to ICU (among non–critically ill patients on day 0), No. (%)	11/78 (14.1)	6/77 (7.8)	1.87 (.67–5.22)	.21
DAF ICU—first 28 d, median (IQR)	25 (18–28)	26 (20–28)	−1.0 (−3.1 to 1.1)	.56
ICU LOS, d, median (IQR)				
Among nonsurvivors	22 (14–31)	15 (10–22)	…	…
Among survivors	2 (0–7)	1 (0–6)	…	…
Hospital LOS, d (among survivors), median (IQR)^d^	10 (6–19)	7 (4–13)	…	…
Patients who met various ARDS oxygenation criteria^e^, No. (%)				
Mild ARDS (PaO_2_/FiO_2_ <300)	77/77 (100)	74/75 (98.7)	1.14 (1.65–.48)	.49
Moderate ARDS (PaO_2_/FiO_2_ <200)	73/77 (94.8)	73/75 (97.3)	0.56 (.11–2.72)	.47
Severe ARDS (PaO_2_/FiO_2_ <100)	43/77 (55.8)	48/75 (64)	0.72 (.37–1.37)	.31
Minimum PaO_2_/FiO_2_ (first 14 d; among critically ill patients on day 0), median (IQR)	84.6 (60.0–124.0)	84.3 (62.0–108.9)	0.3 (−18.8 to 19.4)	.97
No.	77	75		

Abbreviations: ARDS, acute respiratory distress syndrome; CI, confidence interval; DAF, days alive and free; ECMO, extracorporeal membrane oxygenation; ICU, intensive care unit; IQR, interquartile range; LOS, length of stay; NC, not computed (as the majority of the data were 14); PaO_2_/FiO_2_, partial pressure of oxygen/fraction of inspired oxygen; RRT, renal replacement therapy.

^a^Estimated effect of losartan compared to usual care was expressed as odds ratio for binary outcomes and difference in median for count and continuous outcomes.

^b^For those who discharged alive and were subsequently lost to follow-up, they were assumed to be survivors (n = 4 and 8 for 28-day, 12 and 15 for 90-day, and 26 and 34 for 180-day in the 2 arms). However, the discharge and mortality status cannot be ascertained for 4 patients who withdrew consent prior to discharge. See Kaplan-Meier curves (Figure 2) for the sensitivity analysis that censored patients at the last follow-up time point.

^c^Missing for 1 patient.

^d^See Kaplan-Meier curves (Supplementary Figure 1) for the comparison between groups that incorporated both survivors and nonsurvivors.

^e^We did not have chest X-ray data to define ARDS; however, we did have PaO_2_/FiO_2_ so that we could compare ARDS oxygenation criteria between the losartan and usual care groups.

The Kaplan-Meier time to death curves for losartan and usual care groups did not differ (HR, 1.04 [95% CI, .52–2.11]; Figure 2), nor did time to hospital discharge (difference in median, 2.0 days [95% CI, −.2 to 4.2 days]; Supplementary Figure 1). Time to death did not differ between losartan versus usual care in patients who were or were not in ICU at trial admission (Figure 2).

DAF of organ support and ICU were not different between losartan versus usual care (Table 2) and in regression analyses of primary and secondary outcomes (Supplementary Figure 2).

Subgroup analysis did not reveal any statistically significant differences between losartan and usual care groups (Supplementary Figures 3 and 4), except the losartan group had longer median time to hospital discharge in non-White patients (3.1 days [95% CI, .1–.6 days]) and patients without chronic cardiac disease (2.1 days [95% CI, .2–4.0 days]); median DAF ICU was lower in the losartan group within females (−2.5 days [95% CI, −4.5 to −.6 days]).

SAE rates were significantly higher overall (39.8% vs 27.2%, *P* = .01) in the losartan than the usual care arm (Table 3) because the losartan arm hypotension frequency was double that of the usual care arm (30.4% vs 15.3%, *P* < .001).

**Table 3.** Serious Adverse Events of Patients in the ARBs CORONA II Trial of Losartan Versus Usual Care in Patients Hospitalized for Acute Coronavirus Disease 2019

Variable	All (N = 341)	Losartan (n = 171)	Usual Care (n = 170)	*P* Value
Serious adverse events, No. (%)^a^				
Any of the below	114 (33.5)	68 (39.8)	46 (27.2)	.01
New-onset hypotension	78 (22.9)	52 (30.4)	26 (15.3)	<.001
New-onset hyperkalemia	10 (2.9)	5 (2.9)	5 (2.9)	1.00
New-onset acute kidney injury	16 (4.7)	9 (5.3)	7 (4.1)	.61
Severe hypersensitivity reactions	1 (0.3)	1 (0.6)	0 (0.0)	1.00
Acute respiratory distress syndrome	31 (9.1)	17 (10.0)	14 (8.2)	.57
New atrial fibrillation	5 (1.5)	2 (1.2)	3 (1.8)	1.00
New ventricular tachycardia	0	0	0	
New supraventricular tachycardia	1 (0.3)	1 (0.6)	0 (0.0)	1.00
Cardiac arrest	3 (0.9)	1 (0.6)	2 (1.2)	1.00
Disseminated intravascular coagulation	0	0	0	
New rhabdomyolysis	2 (0.6)	0 (0.0)	2 (1.2)	.50
Acute liver failure	3 (0.9)	1 (0.6)	2 (1.2)	1.00
Stroke	0	0	0	
Acute myocardial infarction	0	0	0	
New heart failure	0	0	0	
Deep venous thrombosis	2 (0.6)	0 (0.0)	2 (1.2)	.50
Pulmonary embolism	10 (2.9)	2 (1.2)	8 (4.7)	.05
Peripheral limb embolism	0	0	0	
Other	26 (7.6)	15 (8.8)	11 (6.5)	.41

^a^Data missing for 1 patient.

The overall (28.29% and 14.3%, *P* = .03) and hypotension (17.9% and 3.9%, *P* = .005) SAE rates were also higher for losartan versus usual care in the non-ICU subgroup; hypotension was more frequent (40.9% and 24.7%, *P* = .02) in the ICU subgroup (Table 4). There was no difference between losartan and usual care arms in hypotension SAE rates according to underlying hypertension and no differences in any other SAEs (Table 4).

**Table 4.** Serious Adverse Events of Patients in the ARBs CORONA II Trial of Losartan Versus Usual Care of Patients in Intensive Care Unit (ICU) or Not in ICU on Day 0 in Patients Hospitalized for Acute Coronavirus Disease 2019

Variable	Among Those Not in ICU on Day 0				Among Those in ICU on Day 0			
	All (N = 155)	Losartan (n = 78)	Usual Care (n = 77)	*P* Value	All (n = 186)	Losartan (n = 93)	Usual Care (n = 93)	*P* Value
Serious adverse events, No. (%)^a^								
Any of the below	33 (21.3)	22 (28.2)	11 (14.3)	.03	81 (43.8)	46 (49.5)	35 (38.0)	.12
New-onset hypotension	17 (11.0)	14 (17.9)	3 (3.9)	.005	61 (32.8)	38 (40.9)	23 (24.7)	.02
New-onset hyperkalemia	1 (0.6)	0 (0.0)	1 (1.3)	1.00	9 (4.8)	5 (5.4)	4 (4.3)	1.00
New-onset acute kidney injury	0	0	0		16 (8.6)	9 (9.7)	7 (7.5)	.60
Severe hypersensitivity reactions	0	0	0		1 (0.5)	1 (1.1)	0 (0.0)	1.00
ARDS	6 (3.9)	4 (5.2)	2 (2.6)	.68	25 (13.4)	13 (14.0)	12 (12.9)	.83
New atrial fibrillation	1 (0.6)	0 (0.0)	1 (1.3)	1.00	4 (2.2)	2 (2.2)	2 (2.2)	1.00
New ventricular tachycardia	0	0	0		0	0	0	
New supraventricular tachycardia	0	0	0		1 (0.5)	1 (1.1)	0 (0.0)	1.00
Cardiac arrest	0	0	0		3 (1.6)	1 (1.1)	2 (2.2)	1.00
Disseminated intravascular coagulation	0	0	0		0	0	0	
New rhabdomyolysis	0	0	0		2 (1.1)	0 (0.0)	2 (2.2)	.50
Acute liver failure	2 (1.3)	0 (0.0)	2 (2.6)	.50	1 (0.5)	1 (1.1)	0 (0.0)	1.00
Stroke	0	0	0		0	0	0	
Acute myocardial infarction	0	0	0		0	0	0	
New heart failure	0	0	0		0	0	0	
Deep venous thrombosis	1 (0.6)	0 (0.0)	1 (1.3)	1.00	1 (0.5)	0 (0.0)	1 (1.1)	1.00
Pulmonary embolism	2 (1.3)	1 (1.3)	1 (1.3)	1.00	8 (4.3)	1 (1.1)	7 (7.5)	.06
Peripheral limb embolism	0	0	0		0	0	0	
Other	10 (6.5)	8 (10.4)	2 (2.6)	.10	16 (8.6)	7 (7.5)	9 (9.7)	.60

Abbreviations: ARDS, acute respiratory distress syndrome; ICU, intensive care unit.

^a^Data missing for 1 non-ICU patient.

There was a significantly (5-fold) higher hospital mortality rate in patients who had hypotensive SAEs versus those who did not, in both the losartan arm (OR, 5.37 [95% CI, 1.73–16.6]) and the usual care arm (OR, 6.80 [95% CI, 2.00–23.15]). To understand whether clinical markers and losartan dose were associated with hypotension SAEs, we compared the losartan versus usual care groups without hypotension SAEs: there were no differences (Supplementary Table 4). Hypotension SAEs occurred at losartan doses of 0, 25, 50, and 100 mg in 4%, 33%, 18%, and 45%, respectively, at a median 3 days after starting losartan.

## DISCUSSION

In this RCT in adults hospitalized for acute COVID-19 not previously on ARBs or ACE inhibitors, losartan significantly increased the frequency of hypotension but had no effect on mortality.

We emphasize our rigorous safety process composed of independent medical monitors and a data and safety monitoring board, which recommended stopping our trial early. The safety signal of losartan in COVID-19 and non-COVID-19 community-acquired pneumonia (CAP) is likely generalizable. First, our trial was stopped early because of a safety signal in both ward and ICU patients—hypotension—that we confirmed in other trials and in our open cohort of non-COVID-19 CAP. Second, several other trials [25, 26] found increased risk of hypotension. Meta-analyses of losartan in COVID trials found a numerically increased risk of hypotension [21, 25] and use of vasopressors [26]. Third, the REMAPCAP RCT in COVID-19 of ARBs was stopped for safety issues because of both increased vasopressor use and acute kidney injury [27]. Fourth, in our multicenter observational cohort of patients admitted for non-COVID-19 CAP, patients on ARBs prior to admission had significantly increased vasopressor use and acute kidney injury (28% vs 14%, *P* < .001; J. A. Russell, personal communication).

A recent meta-analysis of RCTs of ARBs in COVID-19 found no mortality benefit but found a trend to increased risk of hypotension in severe COVID-19 (33.8% vs 20.3%; relative risk, 1.56, *P* = .13), especially in trials like ours that introduce de novo ARBs [21]. Hypotension risk was increased in both non-ICU and ICU patients in our trial. Our trial and the meta-analysis [21] suggest some caution for use of ARBs in hospitalized patients in whom ARBs are commonly prescribed (eg, hypertension, 59% patients receive ARBs [28]; hypertensive patients with chronic kidney disease, 35% [29]; diabetics, 32% [30]; and heart failure, 52% [31]).

Our futility analysis assumed no randomization in the ICU and showed less than 7% chance of a significant result in either direction—even with a planned sample size of 1372. This futility was caused in part by the unexpectedly lower pooled mortality rate—about half expected—likely because of changing SARS-CoV-2 variants of concern, advances in treatment (dexamethasone [1] and anticoagulation [2]), vaccination (although vaccination rate was only about 20% overall), and changes in patient mix during various COVID-19 waves [32] (eg, age, sex, and underlying comorbidities). Dexamethasone and full systemic anticoagulation were used in 96% and 18% of our patients, respectively, with no difference between the losartan and usual care arms.

Recent studies of the systemic angiotensin peptide profile in acute COVID-19 might explain a lack of effect on mortality and an increased risk of hypotension with losartan. Plasma angiotensin II level was not elevated in recent studies of patients with COVID-19 and may even be suppressed due to acute lung injury [33–35]. Pulmonary ACE is the dominant site for conversion of angiotensin I to angiotensin II [33]. Pulmonary endothelial injury can cause a systemic angiotensin peptide profile similar to ACE inhibition, perhaps explaining why trials of de novo ARBs in COVID-19 have been neutral so far and could explain why there was an increased risk of hypotension with losartan use in our trial and a trend with ARBs in a meta-analysis [21].

We found serious safety concerns with use of losartan. The increased rate of hypotension was unexpected (although very plausible) because we took steps to exclude high-risk patients and titrated up the losartan dose to avoid hypotension. Also, there could be bias in the reporting of SAEs, so we took steps to ensure systematic reporting. Although there was no mortality difference, losartan doubled the risk of hypotension across the range of losartan doses about 3 days after starting losartan. Losartan doubled the risk of hypotension (30.4% vs 15.3%) and hypotensive patients had about double the mortality of nonhypotensive losartan-treated patients (11.5% vs 4.3%; Supplementary Table 4), but it did not increase overall mortality because the mortality of losartan-treated nonhypotensive patients was nominally lower (4.3%) than the mean mortality of all losartan-treated patients (6.5%). Furthermore, the mortality of usual care patients who had hypotension (15.4%) was higher than the mortality of losartan-treated patients who had hypotension (11.5%; Supplementary Table 4).

We could not identify any markers of risk of hypotension. Hypotension may have occurred in acute COVID-19 because of the vasodilating actions of losartan in the setting of acute COVID-19. Patients with hypotensive SAEs had a much higher mortality rate than patients without such events. The increased hypotension risk suggests for the first time serious safety concerns with use of ARBs in patients hospitalized for COVID-19. ARBs should probably not be added to usual care in such patients.

In 4 RCTs of ARBs in patients hospitalized for acute COVID-19 [14–17] who had not been on ARBs prior to admission, telmisartan significantly decreased mortality compared to usual care [14], whereas 3 other small trials found no effect of ARBs on mortality. One trial found that losartan use was associated with fewer vasopressor-free days than usual care [17]. Other RCTs to continue or discontinue ARBs in patients hospitalized for acute COVID-19 showed no differences in primary outcomes [19, 20]. Hypotension and other safety concerns with ARBs were not found in other trials, perhaps because losartan dosing differed (eg 50 mg twice per day in 1 trial [17] vs 100 mg daily in our trial). Subsequently, recommendations were made to continue ARBs in patients admitted for acute COVID-19 who are already on those drugs [36].

Strengths of our trial are the multisite, 2-country, randomized design enhancing generalizability; the rigorous protocol; the safety monitoring by independent medical monitors and an independent DSMC; and the careful losartan dosing titration regimen.

Limitations are that our trial was stopped for safety concerns and for futility; mortality was lower than expected, only 6%. A lower-than-expected mortality rate might be due to new interventions or changes in SARS-CoV-2 virulence according to variant. We do not have information about SARS-CoV-2 variants; however, our trial was done prior to the Omicron variant. Furthermore, there was lack of placebo so use of open-label losartan could have influenced interpretation of SAEs, but we repeatedly communicated the need to record SAEs in the losartan and usual care arms. Other ARBs could have been more effective than losartan. The losartan dose might not have been optimal; these losartan doses are considered safe and are usual doses for other conditions. Higher doses could have been more effective but less safe.

In conclusion, our trial and the literature indicate serious safety issues with losartan and other ARBs used in patients hospitalized for pneumonia. Caution is needed in deciding which patients hospitalized with pneumonia should use ARBs to mitigate safety concerns, especially hypotension and acute kidney injury. Losartan compared to usual care in patients who had not been on ARBs previously, who were hospitalized for acute COVID-19, did not alter mortality or vital organ function.

## Supplementary Data

Supplementary materials are available at *Clinical Infectious Diseases* online. Consisting of data provided by the authors to benefit the reader, the posted materials are not copyedited and are the sole responsibility of the authors, so questions or comments should be addressed to the corresponding author.

## Supplementary Material

ciae306_Supplementary_Data

## Notes

***Author contributions.*** Conception and design: J. A. R. and K. C. T. Analysis: T. L. and J. S. Interpretation: All authors. Drafting the manuscript for important intellectual content: All authors.

***Acknowledgments.*** The authors thank the patients and their families for giving permission to include them and to report this trial. They also thank the many caregivers who worked tirelessly through the coronavirus disease 2019 (COVID-19) pandemic to provide care to the patients included herein. They also thank the funding agencies.

***Financial support***. This work was supported by the Canadian Institutes of Health Research (CIHR) (grant numbers RN420682 to J. A. R.).
