# Supplementary material for: Effects of Losartan on Patients Hospitalized for Acute COVID-19: A Randomized Controlled Trial
Source: Clin Infect Dis. 2024 Jul 10;79(3):615–25. doi: 10.1093/cid/ciae306 (PMC11426262; doi:10.1093/cid/ciae306)
Supplement: ciae306_Supplementary_Data [file ciae306_supplementary_data.docx]

A**PPENDIX**

Table S1. Trial information and participant disposition of all patients and according to treatment arm in ARBs CORONA II trial of losartan versus usual care in patients hospitalized for acute COVID-19.

|  | **All (n=341)** | **Losartan (n=171)** | **Usual care (n=170)** |
| --- | --- | --- | --- |
| Losartan permanently discontinued, n (%) |  |  |  |
| Unknown |  | 3 |  |
| No |  | 102 (60.7) |  |
| Yes, reason: |  |  |  |
| Subject requests to discontinue study drug |  | 10 (6.0) |  |
| Occurrence of any medical condition or circumstance that exposes the patient to substantial risk and/or does not allow the patient to adhere to the requirements of the protocol |  | 5 (3.0) |  |
| Serious adverse event (SAE) of interest^1^ |  | 34 (20.2) |  |
| Other medical condition deemed related to the study drug |  | 1 (0.6) |  |
| Other^2^ |  | 16 (9.5) |  |
| Maximum losartan dose administered (mg), n (%) |  |  |  |
| Unknown |  | 1 |  |
| 0 (never administered) |  | 10 (5.9) |  |
| 25 |  | 19 (11.2) |  |
| 50 |  | 30 (17.6) |  |
| 100 |  | 111 (65.3) |  |
| Completed protocol, n (%) |  |  |  |
| Yes | 242 (71.0) | 125 (73.1) | 117 (68.8) |
| Still on study/yet to confirm | 22 (6.5) | 12 (7.0) | 10 (5.9) |
| No, reason: |  |  |  |
| Serious Adverse Event(s) | 1 (0.3) | 1 (0.6) | 0 (0.0) |
| Participant request/participant withdrew consent | 11 (3.2) | 7 (4.1) | 4 (2.4) |
| Lost to follow up | 32 (9.4) | 8 (4.7) | 24 (14.1) |
| Death | 29 (8.5) | 14 (8.2) | 15 (8.8) |
| Other | 4 (1.2) | 4 (2.3) | 0 (0.0) |

1. Acute kidney injury (n=2), hyperkalemia (n=3) and hypotension (n=29)

2. Losartan was never administered for 10 patients

- Inadvertent enrollment - prior use of angiotensin receptor blockers or angiotensin converting enzyme inhibitors (n=3)
- Patient request (n=5)
- Discharged prior to first dose (n=1)
- Losartan treatment not administered due to borderline blood pressure (n=1)

Table S2. Additional baseline characteristics of patients randomized to losartan versus usual care in the ARBs CORONA II trial of losartan versus usual care in patients hospitalized for acute COVID-19.

| **Variable** | **All (n=341)** | **Losartan (n=171)** | **Usual care (n=170)** |
| --- | --- | --- | --- |
| Medications pre-admission (within 7 days prior to admission), n (%)^ |  |  |  |
| Antibiotics | 61 (18.0) | 34 (20.0) | 27 (16.0) |
| Steroids | 66 (19.4) | 30 (17.6) | 36 (21.2) |
| Inhaler Steroid | 47 (13.9) | 22 (13.1) | 25 (14.7) |
| Oral Steroid | 17 (5.0) | 7 (4.2) | 10 (5.9) |
| Antivirals | 8 (2.4) | 5 (2.9) | 3 (1.8) |
| HIV | 1 (0.3) | 1 (0.6) | 0 (0.0) |
| COVID-19 | 1 (0.3) | 0 (0.0) | 1 (0.6) |
| Antihypertensive | 51 (15.0) | 25 (14.7) | 26 (15.4) |
| Calcium channel blockers | 29 (8.6) | 8 (4.7) | 21 (12.4) |
| Beta blockers | 28 (8.3) | 16 (9.4) | 12 (7.1) |
| Diuretics | 12 (3.5) | 9 (5.3) | 3 (1.8) |
| Mineralocorticoid receptor antagonists | 1 (0.3) | 1 (0.6) | 0 (0.0) |
| Diabetes insulin | 24 (7.1) | 9 (5.3) | 15 (8.9) |
| Diabetes Oral hypoglycemic agents | 48 (14.2) | 21 (12.4) | 27 (16.0) |
| Metformin | 39 (11.5) | 16 (9.4) | 23 (13.6) |
| Sulfonylurea | 10 (2.9) | 5 (2.9) | 5 (3.0) |
| DPP4 inhibitors | 8 (2.4) | 4 (2.4) | 4 (2.4) |
| SGLT-2 inhibitors | 2 (0.6) | 1 (0.6) | 1 (0.6) |
| GLP-1 receptor agonist | 4 (1.2) | 2 (1.2) | 2 (1.2) |
| Anticoagulants | 64 (18.9) | 38 (22.4) | 26 (15.4) |
| Vitamin K antagonist | 3 (0.9) | 2 (1.2) | 1 (0.6) |
| Direct oral anticoagulant | 13 (3.8) | 8 (4.7) | 5 (3.0) |
| Low molecular weight heparin | 42 (12.4) | 25 (14.7) | 17 (10.1) |
| Heparin | 6 (1.8) | 3 (1.8) | 3 (1.8) |
| Statins | 47 (13.9) | 22 (12.9) | 25 (14.8) |
| Atorvastatin | 20 (5.9) | 8 (4.7) | 12 (7.1) |
| Rosuvastatin | 22 (6.5) | 13 (7.6) | 9 (5.3) |
| Simvastatin | 5 (1.5) | 2 (1.2) | 3 (1.8) |
| sBP, mean (SD)^ | 118.6 (17.8) | 118.1 (16.8) | 119.0 (18.8) |
| dBP, mean (SD)^ | 69.2 (11.8) | 69.0 (12.2) | 69.4 (11.5) |
| Potassium (mmol/L), median (IQR)^ | 4.0 (3.7, 4.3) | 4.0 (3.7, 4.2) | 4.0 (3.7, 4.4) |
| eGRF, median (IQR) | 97.0 (87.0, 113.2) | 95.5 (86.0, 114.2) | 99.0 (91.0, 113.0) |
| # missing | 105 | 51 | 54 |
| PaO2 (mm Hg), median (IQR) | 68 (60, 78) | 67 (58, 77) | 70 (61, 82) |
| # missing | 163 | 78 | 85 |
| FiO2 (%), median (IQR) | 60 (40, 80) | 60 (44, 80) | 57 (40, 80) |
| # missing | 142 | 68 | 74 |
| ALT (U/L), median (IQR) | 51.0 (31.0, 81.0) | 49.0 (31.3, 77.0) | 54.0 (31.0, 86.0) |
| # missing | 38 | 19 | 19 |
| AST (U/L), median (IQR) | 55.0 (39.0, 81.0) | 54.5 (38.5, 79.5) | 55.0 (39.0, 85.5) |
| # missing | 81 | 39 | 42 |
| World Health Organization scale on day 0, n (%) |  |  |  |
| Unknown | 1 | 1 | 0 |
| 3 | 34 (10.0) | 20 (11.8) | 14 (8.2) |
| 4 | 202 (59.4) | 99 (58.2) | 103 (60.6) |
| 5 | 53 (15.6) | 29 (17.1) | 24 (14.1) |
| 6 | 36 (10.6) | 15 (8.8) | 21 (12.4) |
| 7 | 15 (4.4) | 7 (4.1) | 8 (4.7) |
| Medications on day 0, n (%) ^ |  |  |  |
| Antivirals | 19 (5.6) | 10 (5.9) | 9 (5.3) |
| Remdesivir | 13 (3.8) | 7 (4.1) | 6 (3.5) |
| Other | 5 (1.5) | 3 (1.8) | 2 (1.2) |
| Steroids | 300 (88.5) | 147 (87.0) | 153 (90.0) |
| Dexamethasone | 296 (87.3) | 145 (85.8) | 151 (88.8) |
| Hydrocortisone | 2 (0.6) | 1 (0.6) | 1 (0.6) |
| Methylprednisolone | 2 (0.6) | 0 (0.0) | 2 (1.2) |
| Prednisone | 2 (0.6) | 1 (0.6) | 1 (0.6) |
| Other | 3 (0.9) | 2 (1.2) | 1 (0.6) |
| Other Drugs for COVID-19 | 98 (29.0) | 56 (33.1) | 42 (24.9) |
| Antibiotics | 15 (4.4) | 9 (5.3) | 6 (3.6) |
| Anticoagulants | 42 (12.4) | 25 (14.8) | 17 (10.1) |
| Antibody IL6R | 54 (16.0) | 28 (16.6) | 26 (15.4) |
| Antibody spike protein | 0 | 0 | 0 |

^ Missing for up to 4 patients.

Table S3. Additional secondary outcomes in the ARBs CORONA II trial of losartan versus usual care in acute COVID-19 of all patients and according to losartan versus usual care arm in patients hospitalized for acute COVID-19.

| **Variable** | **All (n=341)** | **Losartan (n=171)** | **Usual care (n=170)** | **P** |
| --- | --- | --- | --- | --- |
| Organ support during hospitalization, n (%) |  |  |  |  |
| Oxygen therapy | 300/331 (90.6) | 154/166 (92.8) | 146/165 (88.5) | 0.18 |
| Ventilation | 154/335 (46.0) | 77/166 (46.4) | 77/169 (45.6) | 0.88 |
| Invasive ventilation | 97/332 (29.2) | 49/164 (29.9) | 48/168 (28.6) | 0.79 |
| Non-invasive ventilation | 108/322 (33.5) | 57/161 (35.4) | 51/161 (31.7) | 0.48 |
| RRT | 9/319 (2.8) | 6/158 (3.8) | 3/161 (1.9) | 0.33 |
| ECMO | 9/319 (2.8) | 5/157 (3.2) | 4/162 (2.5) | 0.78 |
| DAF vasopressors - first 14 days, n (%) |  |  |  | 0.94 |
| Unknown | 1 | 1 | 0 |  |
| 0 | 10 (2.9) | 5 (2.9) | 5 (2.9) |  |
| 1-3 | 6 (1.8) | 4 (2.4) | 2 (1.2) |  |
| 4-6 | 4 (1.2) | 2 (1.2) | 2 (1.2) |  |
| 7-10 | 29 (8.5) | 16 (9.4) | 13 (7.6) |  |
| 11-13 | 22 (6.5) | 12 (7.1) | 10 (5.9) |  |
| 14 | 269 (79.1) | 131 (77.1) | 138 (81.2) |  |
| DAF invasive ventilation - first 14 days, n (%) |  |  |  | 1.00 |
| Unknown | 1 | 1 | 0 |  |
| 0 | 35 (10.3) | 17 (10.0) | 18 (10.6) |  |
| 1-3 | 15 (4.4) | 8 (4.7) | 7 (4.1) |  |
| 4-6 | 16 (4.7) | 8 (4.7) | 8 (4.7) |  |
| 7-10 | 22 (6.5) | 12 (7.1) | 10 (5.9) |  |
| 11-13 | 11 (3.2) | 5 (2.9) | 6 (3.5) |  |
| 14 | 241 (70.9) | 120 (70.6) | 121 (71.2) |  |
| DAF RRT - first 14 days, n (%) |  |  |  | 0.84 |
| Unknown | 1 | 1 | 0 |  |
| 0 | 9 (2.6) | 4 (2.4) | 5 (2.9) |  |
| 1-13 | 6 (1.8) | 4 (2.4) | 2 (1.2) |  |
| 14 | 325 (95.6) | 162 (95.3) | 163 (95.9) |  |
| DAF first 14 days (-1 as penalty for death), median (IQR)^ |  |  |  |  |
| Vasopressors | 14 (14, 14) | 14 (14, 14) | 14 (14, 14) | 0.36 |
| Invasive ventilation | 14 (10, 14) | 14 (9, 14) | 14 (10, 14) | 0.93 |
| RRT | 14 (14, 14) | 14 (14, 14) | 14 (14, 14) | 0.80 |
| Medications - first 14 days, n (%) |  |  |  |  |
| Antivirals | 34/339 (10.0) | 18/169 (10.7) | 16/170 (9.4) | 0.70 |
| Remdesivir | 17/339 (5.0) | 9/169 (5.3) | 8/170 (4.7) | 0.79 |
| Other | 10/339 (2.9) | 6/169 (3.6) | 4/170 (2.4) | 0.52 |
| Steroids | 331/339 (97.6) | 163/169 (96.4) | 168/170 (98.8) | 0.17 |
| Dexamethasone | 324/339 (95.6) | 158/169 (93.5) | 166/170 (97.6) | 0.06 |
| Hydrocortisone | 9/339 (2.7) | 7/169 (4.1) | 2/170 (1.2) | 0.10 |
| Methylprednisolone | 13/339 (3.8) | 7/169 (4.1) | 6/170 (3.5) | 0.77 |
| Prednisone | 17/339 (5.0) | 11/169 (6.5) | 6/170 (3.5) | 0.21 |
| Other | 11/339 (3.2) | 8/169 (4.7) | 3/170 (1.8) | 0.12 |
| Other Drugs for COVID-19 | 129/338 (38.2) | 70/169 (41.4) | 59/169 (34.9) | 0.22 |
| Antibiotics | 29/338 (8.6) | 17/169 (10.1) | 12/169 (7.1) | 0.33 |
| Anticoagulants | 61/338 (18.0) | 33/169 (19.5) | 28/169 (16.6) | 0.48 |
| Antibody IL6R | 72/338 (21.3) | 37/169 (21.9) | 35/169 (20.7) | 0.79 |
| Antibody spike protein | 2/338 (0.6) | 1/169 (0.6) | 1/169 (0.6) | 1.00 |
| Medications - while hospitalized or discharged, n (%) |  |  |  |  |
| Antivirals | 48/335 (14.3) | 27/166 (16.3) | 21/169 (12.4) | 0.32 |
| Steroids | 334/339 (98.5) | 165/169 (97.6) | 169/170 (99.4) | 0.22 |
| Antibiotics | 220/335 (65.7) | 112/166 (67.5) | 108/169 (63.9) | 0.49 |
| Antifungal | 18/329 (5.5) | 11/165 (6.7) | 7/164 (4.3) | 0.34 |
| Immune modulator | 116/335 (34.6) | 63/166 (38.0) | 53/169 (31.4) | 0.21 |
| Use of Investigational therapy while hospitalized, n (%) | 14/339 (4.1) | 7/169 (4.1) | 7/170 (4.1) | 0.99 |
| Use of inotropic agents while hospitalized, n (%)^1^ | 4/339 (1.2) | 3/169 (1.8) | 1/170 (0.6) | 0.37 |

1. No troponin and NT-proBNP data was available within 72 hours prior to the use of inotropic agents and thus cannot determine if these patients had acute cardiac injury

^ Missing for 1 patient.

Table S4. Characteristics of patients who did or did not have a hypotension serious adverse event in the losartan and usual care groups in ARBs CORONA II trial of losartan versus usual care in patients hospitalized for acute COVID-19.

|  | **Within Losartan** | | | **Within usual care** | | | |  |
| --- | --- | --- | --- | --- | --- | --- | --- | --- |
|  | **New onset hypotension** | | | **New onset hypotension** | | | |  |
| **Variable** | **Yes (n=52)** | **No (n=118)** | **P** | | **Yes (n=26)** | **No (n=143)** | **P** | |
| Mortality, n (%) |  |  |  | |  |  |  | |
| 28-day | 6/52 (11.5) | 5/115 (4.3) | 0.08 | | 4/26 (15.4) | 5/142 (3.5) | 0.01 | |
| In-hospital | 10/51 (19.6) | 5/115 (4.3) | 0.002 | | 6/26 (23.1) | 6/142 (4.2) | 0.001 | |
| Losartan dose when hypotension occurred, n (%) |  |  | - | |  |  | - | |
| Unknown | 1 | - |  | | - | - |  | |
| 0 (Never started losartan) | 2 (3.9) | - |  | | - | - |  | |
| 25 | 17 (33.3) | - |  | | - | - |  | |
| 50 | 9 (17.6) | - |  | | - | - |  | |
| 100 | 23 (45.1) | - |  | | - | - |  | |
| Day when hypotension occurred |  |  | - | |  |  | - | |
| Median (IQR) | 3.0 (1.0, 5.0) | - |  | | 2.0 (0.0, 4.0) | - |  | |
| Range | (0.0, 27.0) | - |  | | (0.0, 11.0) | - |  | |
| # missing | 1 | - |  | | 1 | - |  | |
| Age |  |  | 0.09 | |  |  | 0.12 | |
| Mean (SD) | 60.1 (13.1) | 56.4 (13.1) |  | | 58.9 (10.3) | 54.4 (14.1) |  | |
| Range | (22.0, 83.0) | (23.0, 86.0) |  | | (36.0, 80.0) | (22.0, 87.0) |  | |
| Sex, n (%) |  |  | 0.60 | |  |  | 0.51 | |
| Female | 18 (34.6) | 36 (30.5) |  | | 6 (23.1) | 42 (29.4) |  | |
| Male | 34 (65.4) | 82 (69.5) |  | | 20 (76.9) | 101 (70.6) |  | |
| Co-morbidities, n (%) |  |  |  | |  |  |  | |
| Hypertension | 10 (19.2) | 24 (20.7) | 0.83 | | 6 (23.1) | 23 (16.1) | 0.38 | |
| Chronic cardiac disease | 4 (7.7) | 12 (10.3) | 0.60 | | 5 (19.2) | 5 (3.5) | 0.002 | |
| Chronic kidney disease | 0 (0.0) | 3 (2.6) | 0.55 | | 1 (3.8) | 5 (3.5) | 1.00 | |
| Diabetes | 11 (21.2) | 15 (12.7) | 0.16 | | 6 (23.1) | 30 (21.0) | 0.81 | |
| Chronic pulmonary disease (not asthma) | 4 (7.7) | 5 (4.2) | 0.35 | | 1 (3.8) | 6 (4.2) | 0.93 | |
| Asthma (physician diagnosed) | 3 (5.8) | 9 (7.6) | 0.66 | | 2 (7.7) | 11 (7.7) | 1.00 | |
| Liver disease | 0 (0.0) | 1 (0.9) | 1.00 | | 1 (3.8) | 1 (0.7) | 0.29 | |
| Prior stroke | 1 (1.9) | 2 (1.7) | 1.00 | | 1 (3.8) | 2 (1.4) | 0.40 | |
| Dementia | 1 (1.9) | 1 (0.8) | 0.52 | | 1 (3.8) | 2 (1.4) | 0.40 | |
| Other chronic neurological disorder | 1 (1.9) | 7 (5.9) | 0.26 | | 1 (3.8) | 6 (4.2) | 0.93 | |
| Malignant neoplasm | 3 (5.8) | 5 (4.2) | 0.66 | | 1 (3.8) | 4 (2.8) | 0.57 | |
| Chronic hematologic disease | 2 (3.8) | 4 (3.4) | 1.00 | | 1 (3.8) | 2 (1.4) | 0.40 | |
| AIDS/HIV | 0 (0.0) | 1 (0.8) | 1.00 | | 1 (3.8) | 0 (0.0) | 0.15 | |
| Hypercholesterolemia | 2 (3.8) | 9 (7.6) | 0.36 | | 5 (19.2) | 17 (11.9) | 0.31 | |
| Rheumatologic disorder | 3 (5.8) | 1 (0.8) | 0.09 | | 2 (7.7) | 3 (2.1) | 0.17 | |
| Venous thromboembolism | 4 (7.7) | 2 (1.7) | 0.07 | | 2 (7.7) | 2 (1.4) | 0.11 | |

P value was based on t-test, Chi-square test or Fisher’s exact test as appropriate.

Figures:

Figure S1. Kaplan–Meier curves for time to hospital discharge of patients (all patients, those in ICU and those not in ICU on day of admission to study) in ARBs CORONA II trial of losartan versus usual care in acute COVID-19. The Log-rank test was used to determine statistical significance.

Figure S2. Regression analysis in all patients in ARBs CORONA II trial of losartan versus usual care in acute COVID-19.

Figure S3. Comparison of losartan versus usual care within subgroups defined by patient characteristics through regression analysis.

Figure S4 - Comparison of losartan versus usual care within subgroups defined by comorbidity status through regression analysis.

Figure S1. Kaplan–Meier curves for time to hospital discharge of patients (all patients, those in ICU and those not in ICU on day of admission to study) in ARBs CORONA II trial of losartan versus usual care in patients hospitalized for acute COVID-19.


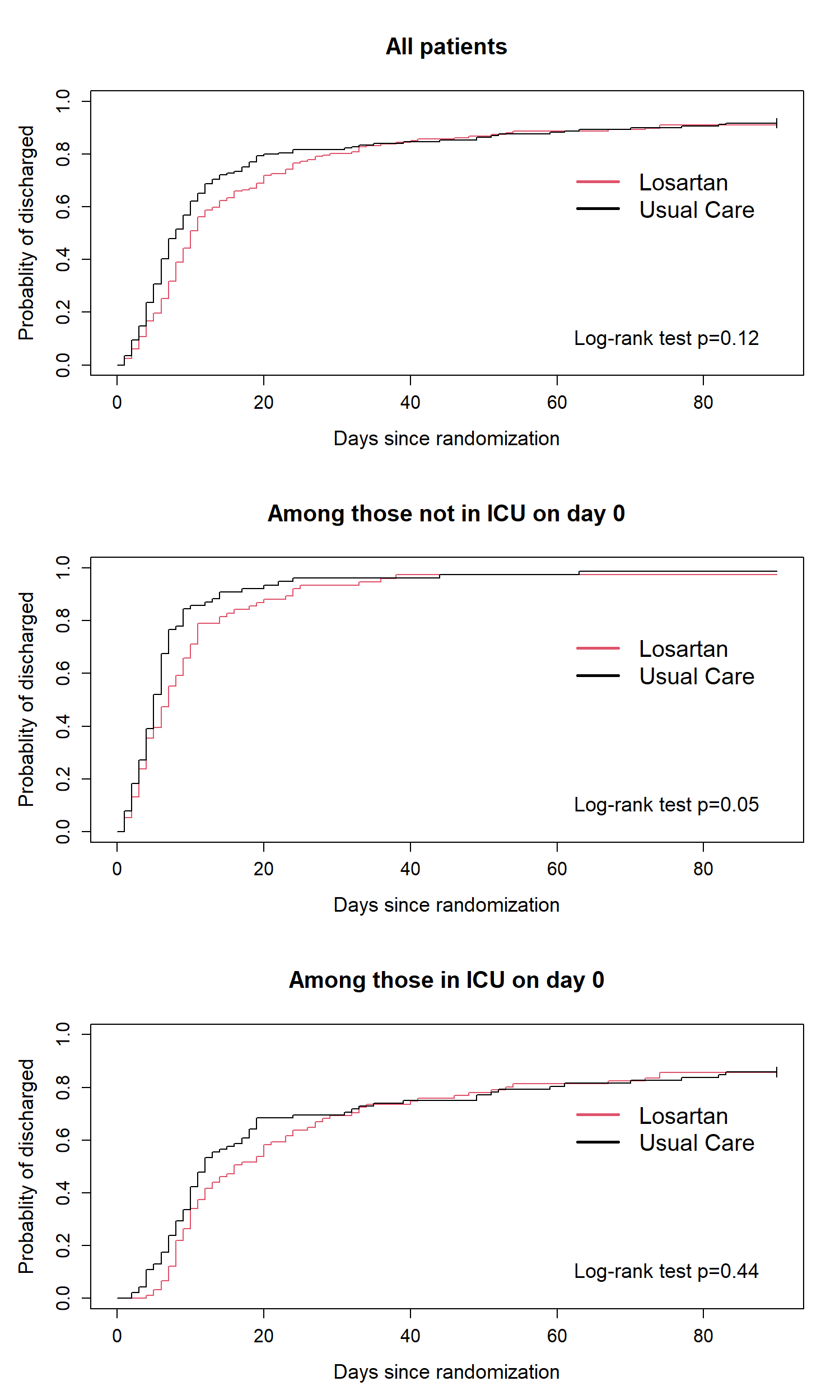


Figure S2. Regression analysis in all patients in ARBs CORONA II trial of losartan versus usual care in patients hospitalized for acute COVID-19.


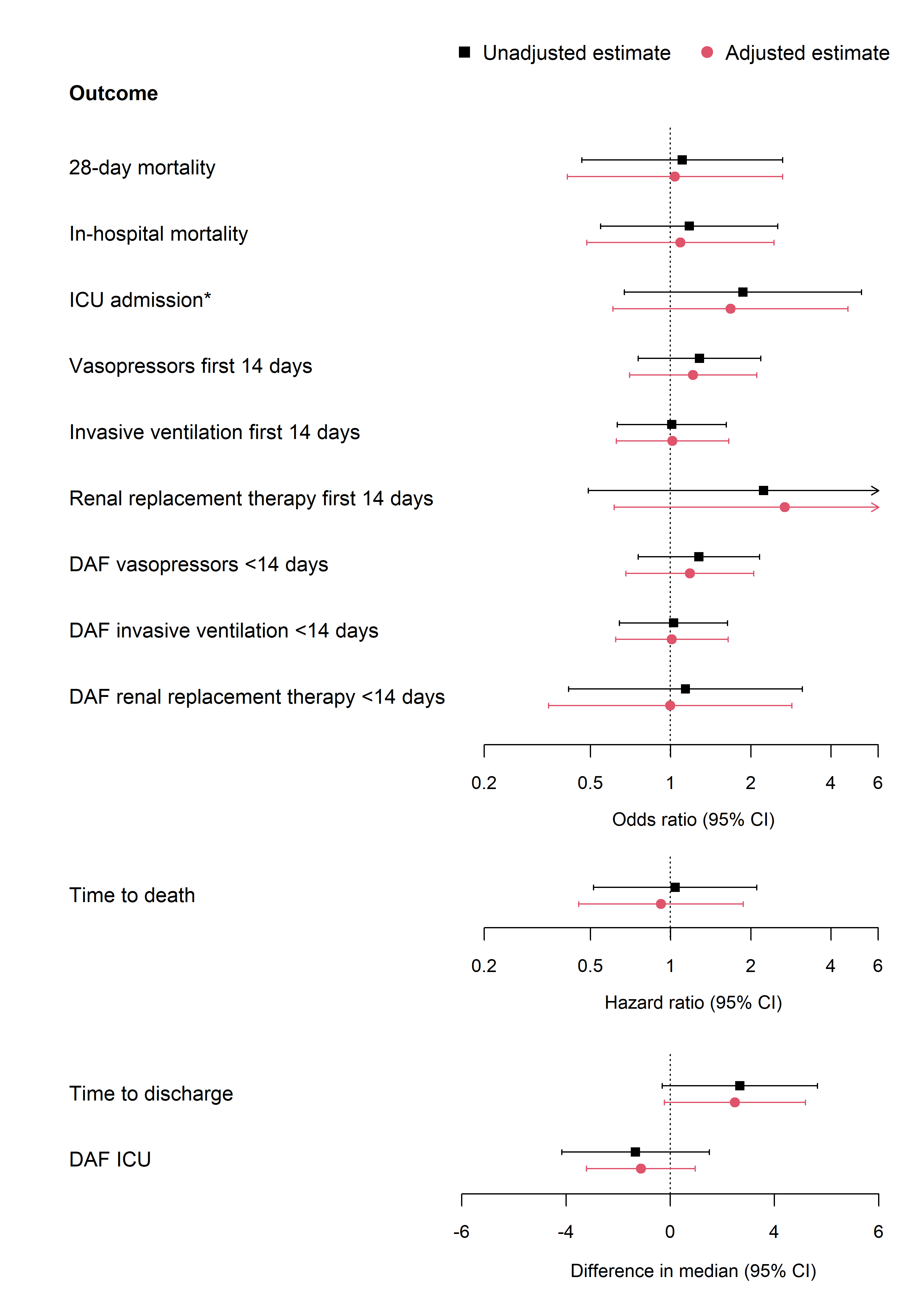


* Within non-critically ill patients on day 0.

Figure S3 - Comparison of losartan versus usual care within subgroups defined by patient characteristics through regression analysis in patients hospitalized for acute COVID-19..

**
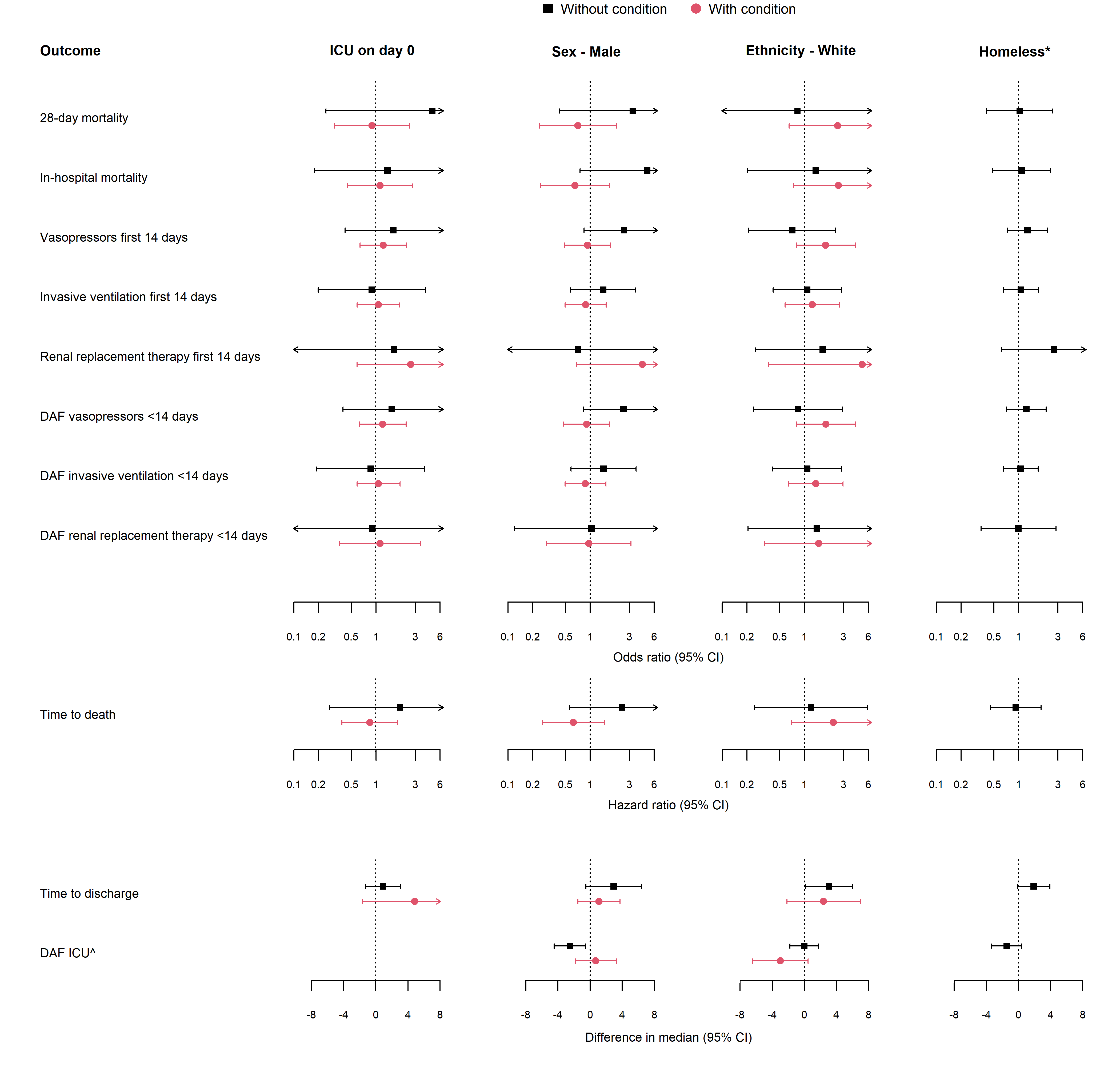
**

^ Not applicable for the subgroup analysis of admitted to ICU on day 0.

* Few patients had the condition and thus analysis was performed only for those without the condition.

Figure S4 - Comparison of losartan versus usual care within subgroups defined by comorbidity status through regression analysis in patients hospitalized for acute COVID-19.


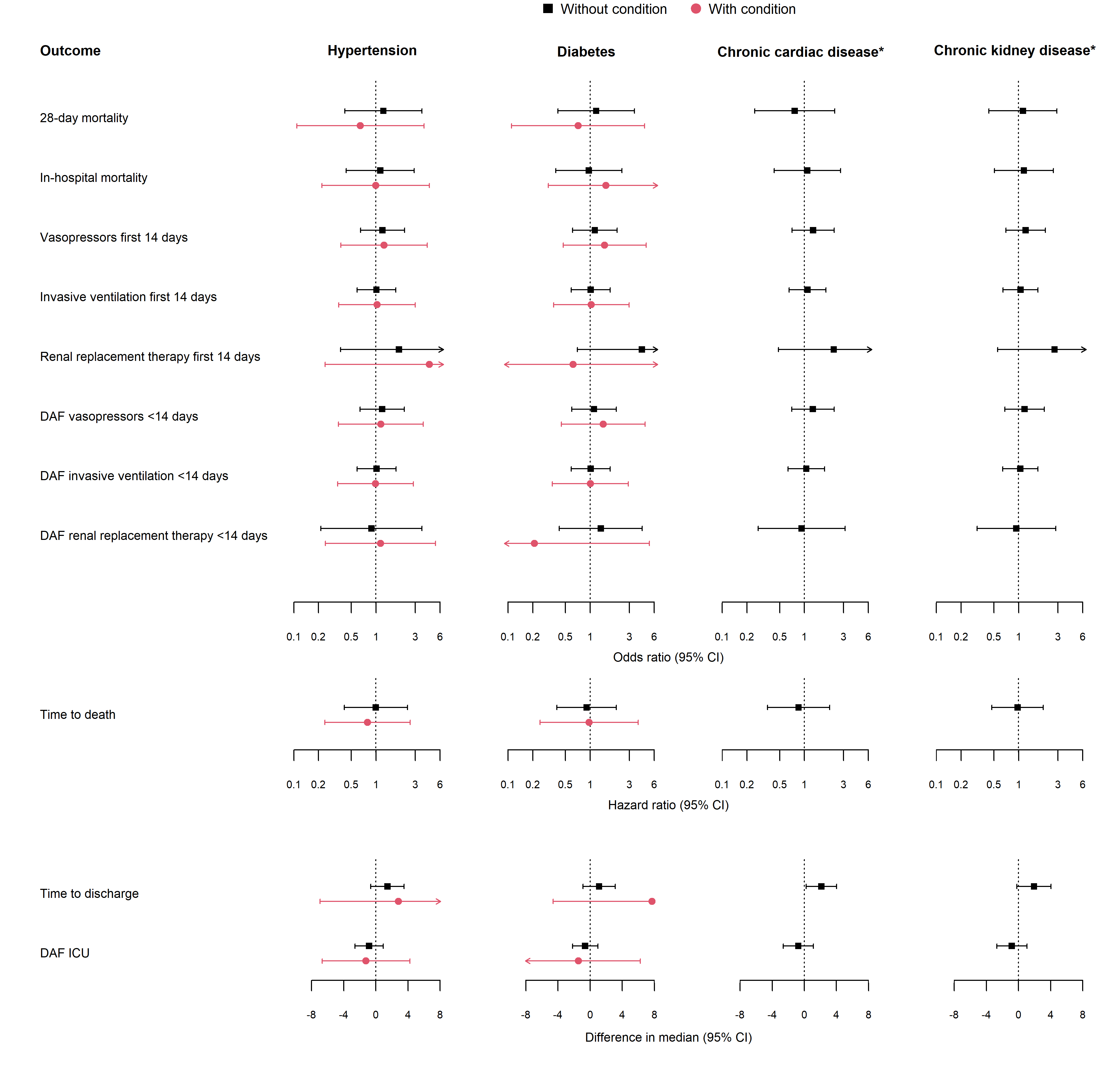


* Few patients had the condition and thus analysis was performed only for those without the condition.

*Randomization*

A permuted blocked randomization method was used with random block size of 2 and 4. A statistician unassociated with the study developed the randomization scheme, consisting of a computer-generated random listing of the treatment allocations in variable permuted blocks. The codes were loaded into an online password-protected web-based allocation program. Upon confirmation of eligibility, sites had 72 hours to randomize patient. When a patient was to be randomized, the study coordinator contacted the pharmacy to confirm eligibility. Someone delegated from the study team used the web-based randomization system to allot assigned treatment to ensure that the randomization scheme was not known by any other site personnel. Upon randomization, site had 12 hours maximum to administer the first dose. An automated audit trail recorded interactions with the system (date and time of allocation, patient identification number and treatment allocation). There was no stratification.

*Blinding maneuvers*

Although use of a placebo would have been better for blinding purposes, given the COVID‑19 issues around speed and feasibility and that (a) the outcome was objective, and thus less susceptible to bias, (b) bias was more likely to be conservative (more co-intervention in the control arm), and (c) we carefully monitored co-interventions continuously to prevent bias, we chose open label design. The coordinating centre statistician was the only person with randomization schedule access. Site pharmacists were responsible for study drug sourcing and preparation in each site hospital pharmacy.

*Losartan Dosing Regimen*

After randomization, patients received losartan 25 mg orally within 12 hours. Assessment for increasing losartan dose occurred after 24 hours. If there were no serious adverse events or AESIs, the dose was increased to losartan 50 mg orally (if the site investigator and/or attending physician had concerns or there was a SAE, losartan dose was maintained at 25 mg). After 24 hours at 50 mg losartan, if there were no serious adverse events or AESIs, losartan dose was increased to 100 mg orally, unless Investigator and/or attending physician discretion dictated otherwise, at which point dosing remained at 50 mg or 25 mg. Subsequent losartan dosing was recommended to occur at the same time each day.

*Discontinuation / Interruption of Losartan*

Losartan could be restarted if the serious adverse event reversed in the judgment of the Investigator and attending physician. Losartan could be discontinued indefinitely if an open label angiotensin receptor blocker or angiotensin converting enzyme inhibitor was started. Losartan could be discontinued or the subject could be removed from the trial for any reason (at the request of the subject or Investigator). Losartan was discontinued if (1) there occurred a medical condition that exposed the patient to substantial risk and/or did not allow the patient to adhere to the protocol, (2) there occurred any serious adverse event, clinically significant adverse event, severe laboratory abnormality, intercurrent illness, or other medical condition deemed related to losartan that indicated to the Investigator that continued participation was not in the best interest of the patient; or (3) a patient failed to comply with protocol requirements or study related procedures.

If a patient withdrew consent or discontinued for any reason, whenever possible, the patient was followed for outcome and safety evaluation. Patients who withdrew or were lost to follow up after randomization were not replaced. Attempts were made to contact patients who were lost to follow up.

*Sequence of Events for Stopping Trial*

The REMAPCAP trial (REF) differed from our trial in that REMAPCAP randomized patients to angiotensin receptor blockers or angiotensin converting enzyme inhibitors versus usual care and had two strata: critically ill and non-critically ill hospitalized patients with acute COVID-19. The sequence of events was the REMAPCAP data were reported to the ARBs CORONA II Principal Investigator; the ARBs CORONA statistician did an unblinded analysis of the primary outcome, rates of acute kidney injury and rates of serious adverse events overall and in subgroups of non-ICU and ICU patients because of the harm of angiotensin receptor blockers in the ICU stratum in REMAPCAP; the DSMC reviewed these results in confidence and then requested a futility analysis; the ARBs CORONA statistician did an unblinded futility analysis of the primary outcome and reported that in confidence to the DSMC; the DSMC recommended that ARBs CORONA II be stopped; this was reported to the ARBs CORONA II Executive Committee that then decided to stop ARBs CORONA II.

*Data Collection and Follow-up*

Baseline data (demographics, clinical standard of care laboratory evaluation, vital signs) for measures of organ function and study drug adherence were collected throughout the treatment period and at 28 days or hospital discharge, if earlier. Patient survival and serious adverse events were documented throughout the treatment period and in the follow up period at 1-, 3- and 6-months post-randomization by in-person and/or virtual clinic visits. The follow up visits included research blood collection (Appendix Protocol and Schedule of Events).

*Missing Data*

For binary mortality outcomes, patients who lost to follow up after being discharged alive were assumed to be survivors at future time points^23^. Sensitivity analysis was conducted using survival analysis technique to censor these patients at the last follow up time point. For DAF organ support, 9 % of patients did not have complete data for the first 14 days and the last known status was carried forward^26^ Some data for organ dysfunction evaluation with SOFA is missing due to death so we planned a separate joint longitudinal and time-to-event model study[49] and inverse probability of censoring weighting[57]).

Text 1.

The losartan doses of 25, 50 and 100 mg have been extensively studied and well tolerated in patients with other conditions including diabetic kidney disease and heart failure. In the RENAAL trial, 71% of patients achieved the maximal losartan dose, and more discontinuations occurred at any time in the placebo arm than the losartan arm (53.5 vs 46.5%), including rare discontinuation due to creatinine elevation (1.5%) or hyperkalemia (1.1%). During the ON-TARGET trial run-in phase, the rapid titration of ramipril plus telmisartan in patients with diabetes and CKD rarely prompted stopping the medications due to hypotension (1.7%) or hyperkalemia (0.8%). The maximal dose of 100 mg per day is indeed less than the 150 mg per day dose used in one arm of the randomized controlled HEAAL trial, which evaluated losartan efficacy in subjects with heart failure [70].

Text 2.

Plasma ACE pathway proteins (ATI, AT1-7, ATII, ACE and ACE2 and metabolomics/proteomics)

We will divide half randomly chosen patients into discovery and the other half validation cohorts and obtain plasma to measure RAS components (ATI, AT1-7, ATII, ACE and ACE2 levels) on days 0, 2, 4, 7 and 14. We will determine: (i) do RAS components predict response to angiotensin receptor blockers? and (ii) what are effects of angiotensin receptor blockers on RAS? The laboratory focuses on ACE2 and will measure RAS components (commercial ELISAs and MS/MS-based assays[51–54]). Metabolomics/proteomics will be measured to determine effects of angiotensin receptor blockers on metabolomics and proteomics. Principle Components Analysis (PCA)[55,56] of RAS proteins and metabolites will be used to increase power. Logistic regression will be used to assess the interaction effect between PCA proteins and metabolites at admission and treatment group on the binary outcomes. To examine metabolites that differentiate treatment groups, (orthogonal) partial least squares discriminate analysis ((O)PLS-DA) will be performed; metabolites with a VIP score > 1.0 will be sought and compared using the model metrics R2 (goodness of fit) and Q2 (goodness of prediction) (Genga submitted). Sensitivities, specificities and area under the receiver operating curve will be determined.

***Cytokines***

There is a growing body of evidence that suggests cytokine storm is associated with more severe COVID-19 infections [60]. It has been postulated that binding of SARS-CoV2 to ACE and upregulation of angiotensin II may trigger cytokine storm. Cytokine testing will be conducted to identify cytokines that may be modulated by ACE pathway. Whole blood will be collected and samples will be analyzed. Further, cytokine panels may be performed depending on findings.

***Genetics (optional)***

For participants who consent to genetics testing, transcriptomic analysis and whole genome sequencing will be conducted to identify candidate genes associated with outcomes and serious adverse events. Whole blood will be collected once for genetic analysis, and PAXgene blood will be collected at multiple time points in alignment with scheduled main study research blood for transcriptomic analysis across disease natural history. Further genetic testing (i.e. targeted panels) may be performed depending on findings.

**Text 3.**

Any unexpected fatal or life-threatening suspected adverse reaction was reported to the regulatory authority as soon as possible, but in no case later than 7 calendar days after initial receipt of the information. If the event was not fatal or life-threatening, the safety report will be submitted within 15 calendar days after the sponsor determines that the information qualifies for reporting. Relevant follow up information to safety report will be submitted as soon as the information is available. Upon request from regulatory authority, the sponsor will submit any additional data or information that the agency deems necessary, as soon as possible, but in no case later than 15 calendar days after receiving the request.

For this study, all AEs, AESIs and unexpected SAEs that occur following initial dose of losartan up to last dose of losartan (or randomization to discharge for those in the standard of care group) will be collected. For those patients attending the 1, 3 and 6 month follow up visits, events will be revisited. Unresolved events at follow up visits will be marked as “ongoing at 1, 3 or 6 month follow up.” There may also be risks that are not known at the present time. Adverse events and serious adverse events will be logged and reported to Health Canada and the local REB as per GCP and ICH Guidance Documents *E6: Guideline for Good Clinical Practice* and *E2A: Clinical Safety Data Management*.

Text 4. Futility analysis.

At the time of the futility analysis, 341 patients were randomized. Data on 28-day mortality was available for 327 patients: 10/162 (6.2%) and 10/165 (6.1%) in the losartan and usual care arm respectively. The pooled 28-day mortality was 2/149 (1.3%) for non-critically ill patients. With the assumption of continue enrolling only non-critically ill patients to reach the final sample size of 1372 and an expected 28-day mortality of 1.25% vs. 1.875% (1/8 of the initial assumption) in the two arms for patients going forward, based on the two-sided conditional power formula stated in the PASS Sample Size Software documentation (Chapter 202; see also Jennison and Turnbull 2000 and Chang 2008), the conditional power would be 0.07.

PASS 2022 Documentation – Conditional Power and Sample Size Reestimation of Tests for the Difference Between Two Proportions. Available at <https://www.ncss.com/software/pass/pass-documentation>. Accessed on March 31, 2022.

Jennison, C., and Turnbull, B.W. 2000. Group Sequential Methods with Applications to Clinical Trials. Chapman & Hall/CRC. New York

Chang, Mark. 2008. Classical and Adaptive Clinical Trial Designs. John Wiley & Sons. Hoboken, New York

**Text 5.** ARBs CORONA II **DATA SAFETY and MONITORING COMMITTEE (DSMC) CHARTER**

| **STUDY TITLE:** | **HOST RESPONSE MEDIATORS IN CORONAVIRUS (COVID-19) INFECTION –**  **IS THERE A PROTECTIVE EFFECT OF LOSARTAN ON OUTCOMES OF CORONAVIRUS INFECTION?**  **(ARBs CORONA II)** |
| --- | --- |
|  |  |
| **PROTOCOL ID:** |  |

**1. OBJECTIVES**

The objectives of the ARBs CORONA II Data Safety and Monitoring Committee (DSMC) will be the following:

1. To make recommendations to ARBs CORONA II Principal Investigator (James Russell) and Steering Committee of Principal Investigators (PI) about the need to modify or terminate clinical trials accepted by the CTN’s Scientific Review Committee (SRC) and SSC;
2. To occasionally review preliminary and interim analyses of COVID-19 study results or new information and recommend changes in protocol, procedures and/or the termination of a study.

**2. MANDATE**

The DSMC’s mandate will be the following:

1. To monitor the conduct and progress of ARBs CORONA II to determine if ARBs CORONA II should be modified or stopped and make recommendations to that effect to the Principal Investigator (James Russell);
2. To evaluate accumulating study data for treatment benefit or harm;
3. To recommend protocol changes;
4. To review and approve clinical trial design and methods insofar as these impinge upon the DSMC’s functions;
5. To alert ARBs CORONA II Principal Investigator (James Russell) and Steering Committee regarding emerging procedural or ethical issues;
6. To monitor enrollment to assure that clinical trials proceed in a timely fashion.

**3. REPORTING**

The DSMC reports to the ARBs CORONA II Principal Investigator (James Russell). The chair of the DSMC transmits DSMC’s recommendations to the ARBs CORONA II Principal Investigator (James Russell).

1. If conflict arises between the DSMC and ARBs CORONA II Principal Investigator (James Russell) on any particular Committee recommendation, a meeting will be held to clarify the conflict and work towards consensus;
2. If the DSMC recommends the termination of a trial for reasons other than relative efficacy, a meeting between the ARBs CORONA II Principal Investigator (James Russell) and the Committee will be arranged. This will help reduce the likelihood that the DSMC would issue a recommendation without being in possession of all the relevant information;
3. In the event of a split vote on the continuance of a trial, the DSMC will report the outcome to the CTN Management Committee (MC) and cite the arguments on either side, the MC will determine the next steps;
4. The DSMC may call in consultants when these are deemed useful in helping the members to achieve an adequate understanding of the issues presented by particular clinical trials;
5. All members of the DSMC have full voting privileges;
6. Any member of the DSMC involved in a study as a treating clinician or co-investigator will be excused from DSMC monitoring data for that study. The DSMC may invite other clinicians on an ad hoc basis to replace those Committee clinicians;
7. Co-investigators who served as methodologists or statisticians may, as DSMC members, participate fully in discussions, but are to exempt themselves from the deliberation phase of closed meetings if their conflict of interest would likely hinder the Committee’s decision process.

**4.** **REVIEWS AND PROCEDURES**

1. The DSMC has a responsibility to review ARBs CORONA II protocol to ensure that the design and methodology of ARBs CORONA II will not hinder the Committee’s ability to assess the relative safety and efficacy of the study treatments;
2. The DSMC’s review of ARBs CORONA II protocol should be done early enough to ensure that its recommended protocol change(s), if any, will not delay or otherwise hinder the clinical trial evaluation process;
3. Prior to the initiation of ARBs CORONA II, the DSMC will strive to meet with ARBs CORONA II Principal Investigator (James Russell) to ensure that the DSMC members have a full understanding of ARBs CORONA II goals and methods, and to ensure that ARBs CORONA II Principal Investigator (James Russell) understands the DSMC’s operating procedures;
4. The DSMC will aim for consensus on its recommendations, but will not insist on unanimity.

**Modification or early termination of a trial**: The following considerations are deemed particularly pertinent to a recommendation for early termination of ARBs CORONA II:

1. ARBs CORONA II may be terminated early if accumulated data indicate that trial participants are being harmed by the treatment;
2. The ARBs CORONA II team will review literature prior to each DSMC meeting and submit summaries and links to published trials of ARBs in COVID-19;
3. Continuation of ARBs CORONA II is justified only so long as there is no way to choose between the test and control treatments;
4. If accumulated data indicate, to the satisfaction of the DSMC, the superiority of one treatment over another, the inferior treatment should be stopped;
5. The costs of ARBs CORONA II are no longer justified once accumulated results allow a reliable discrimination between the treatments;
6. If ARBs CORONA II is no longer likely to produce additional useful information should be stopped;
7. A monitoring committee carries responsibilities not only towards patients currently participating in ARBs CORONA II, but also towards patients scheduled to enter ARBs CORONA II, and to future ARBs CORONA II patients;
8. The DSMC may have to advise ARBs CORONA II Principal Investigator (James Russell) to modify ARBs CORONA II protocol, to terminate ARBs CORONA II ahead of schedule, to continue ARBs CORONA II as planned, or even to extend a ARBs CORONA II beyond the scheduled point of termination;
9. ARBs CORONA II may have to be terminated because of excessively slow enrollment combined with the likelihood that enrollment targets will never be reached on time to allow a clinically meaningful completion of ARBs CORONA II.

**5. ACCESS TO INFORMATION**

The DSMC’s activities will require that members of the committee have access to pertinent information, e.g., ARBs CORONA II research protocols, personnel information and other supporting documents. All such documents will be kept in the strictest confidence.

**6. WORKING DOCUMENTS**

1. The terms of reference for the DSMC will be treated as ‘working’ documents and reviewed regularly as required;
2. The purpose of the terms of reference for the DSMC is to avoid the need to constantly reconstruct discussions and conclusions of previous meetings about how the Committee should operate;
3. The terms of reference of the DSMC are to be used as protection against arbitrariness, inconsistency and unfairness in the Committee’s deliberations and decisions;
4. The terms of reference should be referred to in cases of dispute about how the DSMC should proceed, or whether the Committee is proceeding according to its mandate and responsibilities in specific cases.

**7. CONFIDENTIALITY**

The DSMC maintains responsibility to the ARBs CORONA II Principal Investigator (James Russell) and Steering Committee, CTN, the sponsors, ARBs CORONA II investigators, staff and trial participants that its deliberations and the information to which it becomes privy, be treated with the utmost confidentiality by all its members. All materials viewed by members of the DSMC must be kept in the strictest confidence. These items cannot be circulated outside the DSMC unless otherwise decided by the DSM Committee and ARBs CORONA II Principal Investigator (James Russell) and Steering Committee. Failure to honour confidentiality is grounds for dismissal from the DSMC. Each Committee member will sign a Confidentiality Agreement with the CTN.

**8. REPRESENTATION**

**Membership:** Members of the DSMC should possess the range of disciplines needed to assure that the Committee has the expertise to deal with the complexities of the issues it will preside over.. The membership should also enjoy sufficient stature among peers to maximize general acceptance of its reasoned judgments. Members must be;

1. Free from conflicts of interest;
2. Chosen in such a way as to assure a separation of persons responsible for patient care in the trial from persons responsible for safety and efficacy monitoring.

**Make-up:** It is essential that the DSMC membership should include persons who:

1. Are physicians with wide experience and knowledge regarding the course and treatment of COVID-19 disease;
2. Are experts on statistical and methodological aspects of COVID-19 clinical trials;
3. Are skilled in identifying ethical and legal implications of clinical trials;
4. Understand and accept the general principles and procedures of the clinical trial process.

**9. APPOINTMENTS, THE CHAIR, LENGTH OF TERMS & QUORUM**

**Appointments:** Members are appointed to the DSMC by the MC.

**Chair:**

1. The Chair will be appointed for a designated term not to exceed three years. The chair may be reappointed for an additional term.
2. If the Chair resigns during the term, a successor will be appointed for the time remaining in that term.

**Responsibilities of the Chair:**

1. Providing an annual report of the DSMC’s activities to ARBs CORONA II Principal Investigator (James Russell);
2. Reviewing the agenda and minutes for each meeting;
3. Reporting to the MC;
4. Participating in any review of the DSMC;
5. Chairing meetings and delegating responsibilities.

**Length of terms:**

1. DSMC members will be appointed for three-year terms.
2. To ensure continuity, efforts must be made to ensure that the majority of Committee members do not rotate at one time.
3. Under appropriate circumstance some members may have their appointment extended beyond three years.
4. Members are required to attend meetings. If a member misses three consecutive meetings without an explanation that is acceptable to the DSMC, the member will be asked to step down from the Committee.

**Quorum:** To take decisions or make recommendations, the DSMC requires the presence of at least two thirds of its members, including the presence of at least two clinicians.

**10. MEETINGS**

**Frequency:** The DSMC will meet as stated in the ARBs CORONA II protocol (at 25%, 50% and 75% of recruited patients), with additional meetings via teleconference on an ad hoc basis.

**Other meetings:** The DSMC will meet at any other time when important issues arise, such as unexpected adverse events or the occurrence of new external data that could affect the continuance of a clinical trial as planned.

**11. SECRETARIAT AND FINANCIAL SUPPORT**

The Secretariat for the DSMC will be provided by the CTN. The CTN will provide secretarial support in the form of meeting coordination and other support tasks. The CTN will also take and circulate minutes of meetings to Committee members.

**12. CONFLICT OF INTEREST GUIDELINES**

Members of the DSMC will be subject to the same Conflict of Interest Guidelines that govern the CTN’s other committees. Members who are participating or are involved in the development of a study must absent themselves from Committee discussions of these trials.
